# Improving structural similarity based virtual screening using background knowledge

**DOI:** 10.1186/1758-2946-5-50

**Published:** 2013-12-16

**Authors:** Tobias Girschick, Lucia Puchbauer, Stefan Kramer

**Affiliations:** 1 Technische Universität München, Institut für Informatik, Boltzmannstrasse 3, 85748 Garching b. München, Germany; 2 Johannes Gutenberg-Universität Mainz, Institut für Informatik, Staudingerweg 9, 55128 Mainz, Germany

**Keywords:** Virtual screening, Structural similarity, Background knowledge, Data mining, Enrichment

## Abstract

**Background:**

Virtual screening in the form of similarity rankings is often applied in the early drug discovery process to rank and prioritize compounds from a database. This similarity ranking can be achieved with structural similarity measures. However, their general nature can lead to insufficient performance in some application cases. In this paper, we provide a link between ranking-based virtual screening and fragment-based data mining methods. The inclusion of binding-relevant background knowledge into a structural similarity measure improves the quality of the similarity rankings. This background knowledge in the form of binding relevant substructures can either be derived by hand selection or by automated fragment-based data mining methods.

**Results:**

In virtual screening experiments we show that our approach clearly improves enrichment factors with both applied variants of our approach: the extension of the structural similarity measure with background knowledge in the form of a hand-selected relevant substructure or the extension of the similarity measure with background knowledge derived with data mining methods.

**Conclusion:**

Our study shows that adding binding relevant background knowledge can lead to significantly improved similarity rankings in virtual screening and that even basic data mining approaches can lead to competitive results making hand-selection of the background knowledge less crucial. This is especially important in drug discovery and development projects where no receptor structure is available or more frequently no verified binding mode is known and mostly ligand based approaches can be applied to generate hit compounds.

## Background

Medical needs are the starting point for every drug discovery and development project. Apart from the classical *in vitro* and *in vivo* studies used in this process, pharmaceutical research relies more and more on *in silico* methods like (high throughput) virtual screening or molecular docking simulations [1,2]. Computational methods promise to shorten the typically time-consuming efforts that come with the development of new market-approved drug compounds. In the early drug discovery process, virtual screening is used to rank or select compounds from huge databases of potential drug candidates that are later assessed in wet-lab and animal studies. In case one or more ligand structures of the target protein are known and available, virtual screening based on ligand similarities can be used to calculate a ranking of candidate compounds in a database. This approach is applied if no binding mode of the reported ligands, as well as no X-ray or NMR structure of the protein target is available and receptor based approaches are not easily accessible. Yet even in these cases the virtual screening approach is certainly a valid orthogonal approach to derive interesting and promising structures and scaffolds for the drug discovery pipeline.

In this paper, we present a concept of how structural similarity based methods used in virtual screening can be improved by integrating chemical background knowledge in the form of binding relevant or informative structural elements. Improvement in this case means higher enrichment of chemical compounds related to the query compound in the similarity ranking of a compound database. Consequently, more potentially biologically active and less potentially inactive compounds are selected in virtual screening for further processing in the drug discovery pipeline (e.g. *in vitro*, *in vivo*). To achieve an improved enrichment we extract binding relevant substructures from known ligands and transform them into a fingerprint. This fingerprint is then used to extend a structural similarity measure. We present two approaches to extract the binding relevant information: first we use visual inspection of a known ligand as well as literature review to identify binding relevant substructures, second we test a relatively basic data mining approach. We apply the Free Tree Miner (FTM) software [3] that takes a set of two-dimensional chemical structures as input. FTM mines for and returns all substructures that occur frequently (more often than a user defined minimum support threshold) in the given set. These relevant substructures are then fragmented and the fragments’ occurrences in a chemical structure are used as bits in a binary occurrence fingerprint. A limitation of the data mining based approach is the need for more than one known ligand (active compound). An advantage of the approach is that it can still be applied if no literature information on the binding relevant substructures or structural patterns is available and that it saves human effort.

In our experiments we extend two structural similarity measures with background knowledge and apply them to rank compounds in a database according to their similarity to a known active structure. The first similarity measure is based on the size of the maximum common substructure (MCS – e.g., Raymond *et al.*[4]) of two molecules, the second is based on Extended Connectivity Fingerprints (ECFP) [5]. No other factors like drug-likeness, Cytochrome P450 interaction or physico-chemical properties are used. This enables an isolated view on the effects of the similarity methods used for the rankings. The extended similarity measures are compared to their non-extended versions to assess their performance by calculating enrichment factors for 1%, 5% and 10% of the database.

We show that adding background knowledge on important binding components of ligands to both, the MCS similarity and the ECFP similarity, changes the virtual screening ranking in such a way that the top structures have improved docking scores, related structures are ranked at better positions and clearly improved enrichment factor values are obtained. We also show that replacing the visual inspection and literature search by a data mining approach improves the similarity rankings for most assessed data sets. The data mining approach performs slightly weaker than the by-hand approach, but gives competitive results.

The remainder of the paper is organized as follows: In the next section we give detailed information on the data and methods we use for the similarity calculations and our experimental setup. This is followed by a presentation and discussion of our results before we conclude. Additional result tables can be found in the Additional file 1.

## Materials and methods

In this section we give detailed information on our experimental setting, on how we extend a similarity measure and on the data sources and evaluation measures used in our virtual screening experiments.

### Experimental setup

When virtual screening by means of similarity ranking is performed in a drug discovery project, the similarities of all compounds in the screening database are calculated with respect to one or more known ligands of the protein target (used as reference compounds). The compounds in the database are subsequently sorted according to their similarity scores in descending order so that the compounds most similar to the reference appear first in the ranking. A good similarity measure will find structures that are related to the reference – or that potentially interact with the target protein – in the first few percent of the list. To assess the performance of different similarity measures we mix a set of known ligands into a set of decoys to form a screening database. As reference compound for the similarity rankings we use a randomly selected representative of the known ligands. After applying the standard similarity ranking procedure individually with each similarity measure, we can evaluate the performance of the similarity measures by examining the results for the known ligands in the screening database. The better a similarity measure is, the more known ligands will be in the top section of the ranking.

The experiments on extending a structural similarity measure can be divided into two lines of experiments: line “A” considers the by-hand selection of the binding relevant information that is used to extend the similarity measure and line “B” considers the data mining based selection of this information.

Table 1 shows a comparison of the steps necessary to apply the two presented approaches to extend similarity measures and rank a screening database.

**Table 1:** Overview of the steps necessary to apply the two presented approaches to extend similarities

**Step**	**A: by-hand approach**	**B: mining-based approach**
1	Review literature/examine structure	Calculate frequently occurring
	to determine BI	substructures (BI) in known
		ligands with FTM
2	Fragment relevant substructure	Build fingerprint from
	and build binary occurrence	frequently occurring
	fingerprint from all fragments	substructures
3	Rank DB with *sim*_*e* *x* *t*_	Rank DB with *sim*_*e* *x* *t*_

Overview table of the steps necessary to apply the two presented approaches to extend similarities. DB: database; BI: binding-relevant information.

### Extended similarity

The extended similarity measures proposed in this work are constructed from two building blocks: a structural similarity measure used as base simililarity (*sim*_*b* *a* *s* *e*_) and a fingerprint-based similarity that is based on the binding relevant substructures ($\text{si}m_{\text{bind\_fp}}$). After defining the extended similarity measure we will first explain the base similarities and second explain the two variants used to derive $\text{si}m_{\text{bind\_fp}}$. The *extended similarity* of two molecules *a* and *b* is defined as:

(1) $$\text{si}m_{\text{ext}}\left(a,b\right)=1-\mathrm{\alpha si}m_{\text{base}}\left(a,b\right)+\mathrm{\alpha si}m_{\text{bind\_fp}}\left(a,b\right),$$

where $\text{si}m_{\text{bind\_fp}}\left(a,b\right)$ gives the Tanimoto similarity coefficient (for a mathematical definition see the Additional file 1) of two binary sub-structural occurrence fingerprints of molecules *a* and *b*.

For most experiments we choose $\alpha =\frac{1}{3}$ as weight coefficient for the fingerprint-based part arbitrarily and motivated by the wish to weight the base similarity higher than its extension. No optimization regarding this parameter has been attempted, however we show a short evaluation of *α* in the Results and discussion section. In our experiments the substructures constituting the fingerprint for $\text{si}m_{\text{bind\_fp}}$ are selected by visual inspection and literature review or by a data mining approach.

The first structural similarity measure (*sim*_*b* *a* *s* *e*_) that we extend is based on the notion of maximum common substructures (MCS). For computation of the size of the MCS of two molecular structures, the JChem Java classes were used (JChem 5.4.0.0, ChemAxon (http://www.chemaxon.com)). The similarity between two structures was then calculated with the similarity measure proposed by Wallis *et al.*[6]:

(2) $$\text{si}m_{\text{MCS}}\left(a,b\right)=\frac{\left|\text{mcs}\left(a,b\right)\right|}{\left|a\right|+\left|b\right|-\left|\text{mcs}\left(a,b\right)\right|},$$

where |·| gives the number of vertices in a graph, and *mcs*(*a*,*b*) calculates the MCS of molecules *a* and *b*. Consequently, |*mcs*(*a*,*b*)| is the number of atoms of the MCS of molecules *a* and *b*. The second structural similarity measure is based on Extended-Connectivity Fingerprints (ECFP) [5], a standard method in pharmaceutical research and industry. ECFP fingerprints are circular, structural feature fingerprints that use as input information not only the atom and bond type, but the six atom numbering independent Daylight atomic invariants [7] to encode atoms: the number of immediate heavy atom neighbors, the valence minus the number of hydrogens, the atomic number, the atomic mass, the atomic charge, the number of attached hydrogens, plus a seventh invariant added by Rogers *et al.*[5]: whether the atom is contained in at least one ring. These fingerprints are available via the RDKit functionality of the open source cheminformatics software AZOrange [8]. The radius parameter for the ECFP fingerprint calculation was used at the default value of *r*=2. The fingerprint similarity of two ECFP fingerprints is calculated with the Dice coefficient (for a mathematical definition see the Additional file 1).

Our first approach (approach A) to extend *sim*_*b* *a* *s* *e*_ relies on literature review or visual inspection of a set of known ligands to retrieve a binding relevant substructure (or fragment). Once such a substructure is known we apply the Free Tree Miner [3] software without minimum frequency constraint to produce all possible fragments of the substructure. From these fragments we build a binary occurrence fingerprint that is used to encode the reference molecules and all database molecules. The fingerprints are then used to calculate $\text{si}m_{\text{bind\_fp}}$. In our experimental evaluation of approach A on the HMGR data set, we derive the binding relevant substructure not only by literature review (which would be the standard approach and sufficient in most cases), but we support the process by additional calculations. First, we use the MCS similarity measure to rank the screening database. Subsequently, the top 25 compounds of the similarity ranking are docked to the HMGR receptor. The examination of the results in combination with the literature review is used to derive the binding relevant structural parts that are used as background knowledge. For the second data set used to evaluate approach A (PPAR *γ*) we derive the binding relevant stubstructure from reviewing known ligands from the DrugBank [9] database. We expect the PPAR *γ* hand-selection experiments to show less improvement than those on HMGR as the binding relevant information is selected with less effort.

In our second approach to extend *sim*_*b* *a* *s* *e*_, the data mining based approach - denoted approach B, we try to substitute the by-hand selection of the additional knowledge that is integrated into the similarity measure by applying data mining techniques. To retrieve the substructure fingerprint used for the similarity measure extension we calculate the set of frequently occurring substructures from a set of known ligands with the FTM algorithm. From those frequent substructures we build the binary occurrence fingerprint used to encode our molecules and used to calculate $\text{si}m_{\text{bind\_fp}}$. Two variant of input ligand sets are tested: (B1) We use all available ligands for the generation of the fingerprint fragments. The minimum support parameter (*minsup*) for the FTM software was chosen in such a way for each data set that it resulted in approximately the same number of substructural features as the fingerprint of approach A did (57 features). The parameters are given in Table 2. This ensures that we can exclude the lenght of the fingerprint (feature number) as driving force of improvement or degradation. (B2) We use only 10% (20% in case of the DuD HMGR, ADA and TK data sets) of the ligand compounds randomly chosen from the respective DuD ligand sets to work with a more realistic setting, where only few compounds interacting with the protein are known in advance. The minimum support parameter of FTM was set to 0.9 for all data sets. This second, reduced variant provides less information on the ligands to be found in the ranking and consequently poses a more realistic but harder problem. The resulting enrichment factor values of the extended similarity measures should show less improvement over the non-enxtended versions compared to the first variant that uses all ligands.

**Table 2:** Overview of the used DuD data sets

**Protein**	**PDB code**	**Ligands**	**Decoys**	**Protein class**	***minsup***	***fp_length***
HMGR	[PDB:1HW8]	35	1242	other enzyme	0.9	66
ER	[PDB:3ERT]	39	1399	nuclear hormone receptor	0.7	62
PPAR *γ*	[PDB:1FM9]	81	2910	nuclear hormone receptor	0.96	90
P38 MAP	[PDB:1KV2]	234	8399	kinase	0.83	57
TK	[PDB:1KIM]	22	785	kinase	0.9	74
FXa	[PDB:1F0R]	142	5102	serine protease	0.8	81
ADA	[PDB:1STW]	23	822	metalloenzyme	0.8	70
DHFR	[PDB:3DFR]	201	7150	folate enzyme	0.8	70
AChE	[PDB:1EVE]	105	3732	other enzyme	0.77	93
COX-2	[PDB:1CX2]	349	12491	other enzyme	0.6	65

Overview of the used DuD data sets. *minsup* gives the minimum support parameter used in the FTM calculations and *fp_length* the length of the resulting fingerprint.

**Figure 1:**
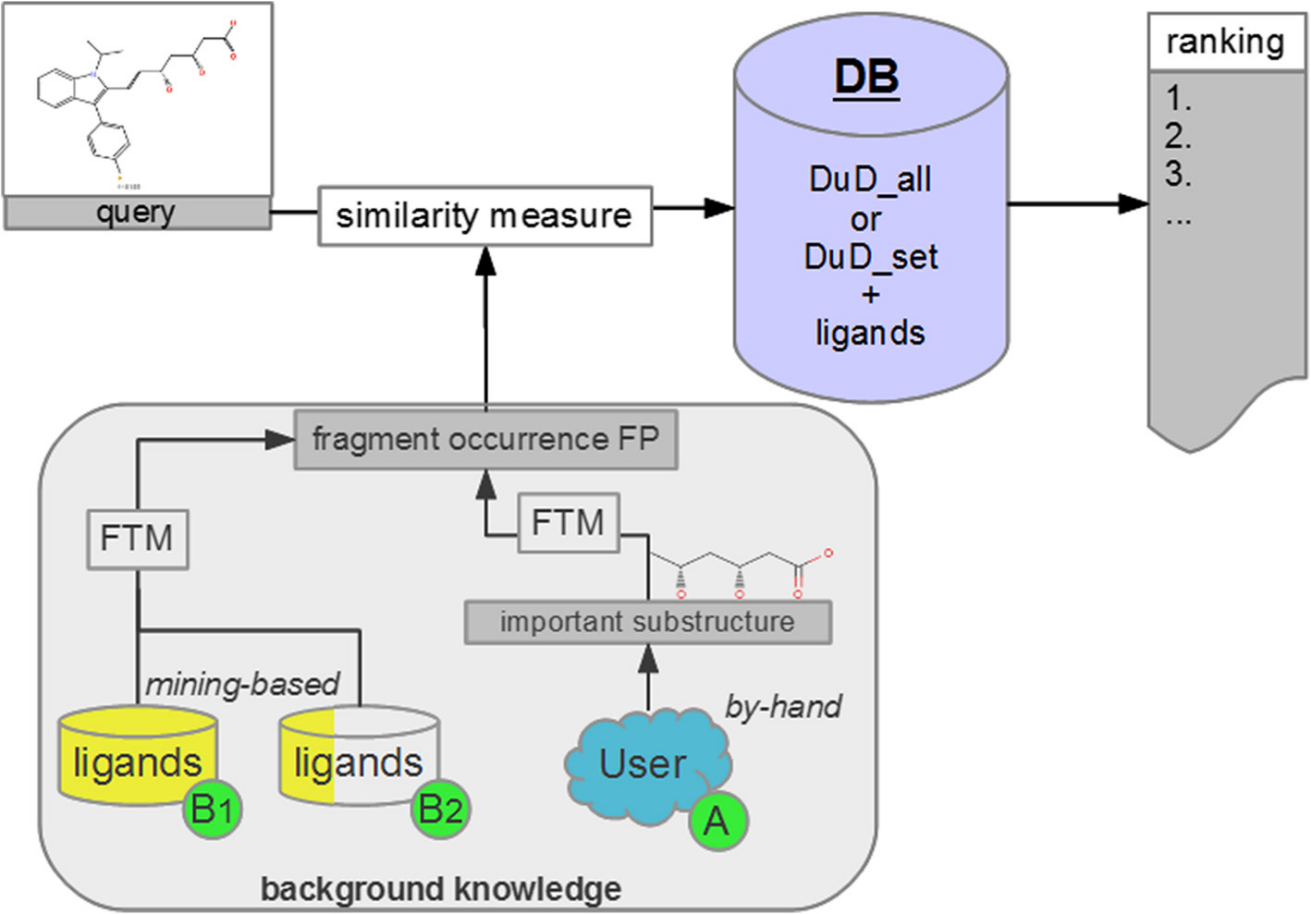
**Overview of the experimental setup of the (A) by-hand extension experiments (B) mining-based extension experiments.** The upper half of the workflow shows a similarity ranking without the incorporation of background knowledge. FP = fingerprint.

For a graphical overview of the two extension approaches as well as how they interact with the base-line similarity ranking please see Figure 1.

### Data

In the first line of experiments (by-hand selection) we use only two data sets for our analysis, in line two of the experiments (data mining based extension) we use ten data sets from the Directory of useful Decoys (DuD) [10] as well as 25 ChEMBL activity class data sets [11]. We use different database setups in our evaluation: For experiments with the DuD data sets we use either all 95,000 decoy structures of the DuD (DuD_*all*_) or only those DuD decoys as database that were designed especially for the target ligand system considered (DuD_*set*_). For the experiments with the ChEMBL activity classes we use a subset of the ZINC [12] database.

#### HMGR and statins

**Figure 2:**
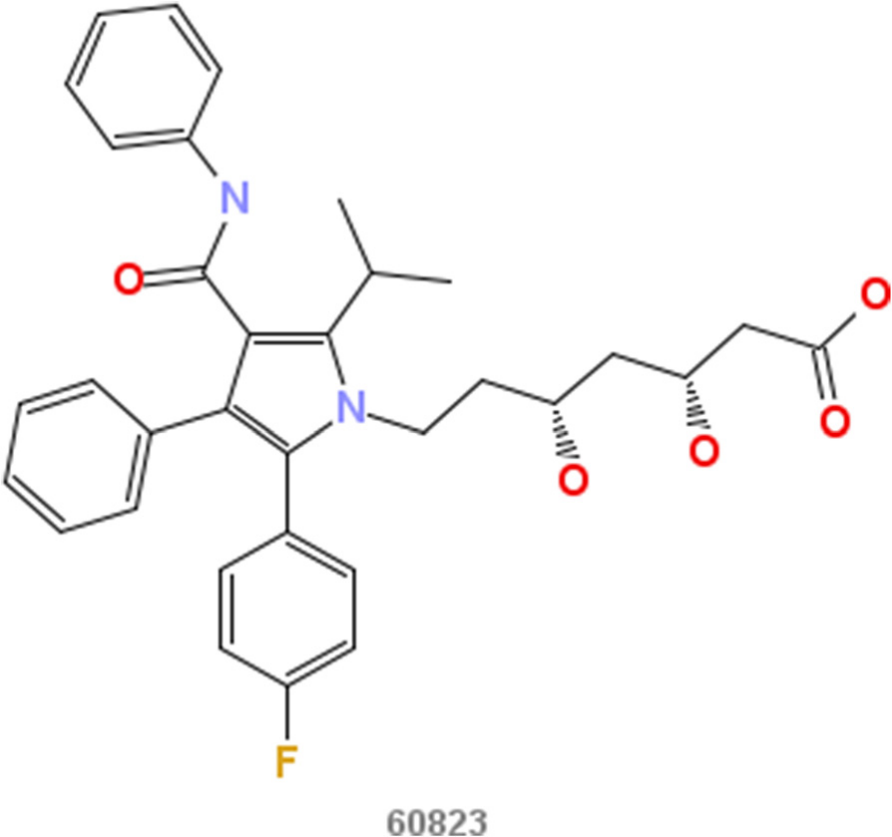
**Atorvastatin.** 2D structure depiction of Atorvastatin (PubChem CID 60823).

**Figure 3:**
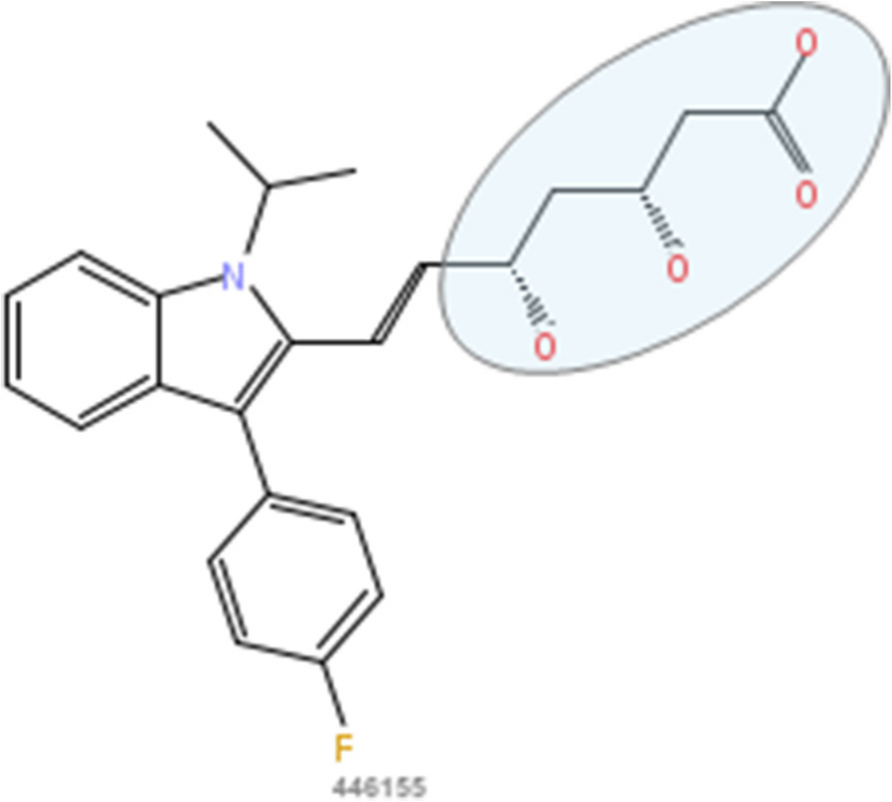
**Fluvastatin.** 2D structure depiction of Fluvastatin (PubChem CID 446155), the binding relevant substructure is marked.

**Figure 4:**
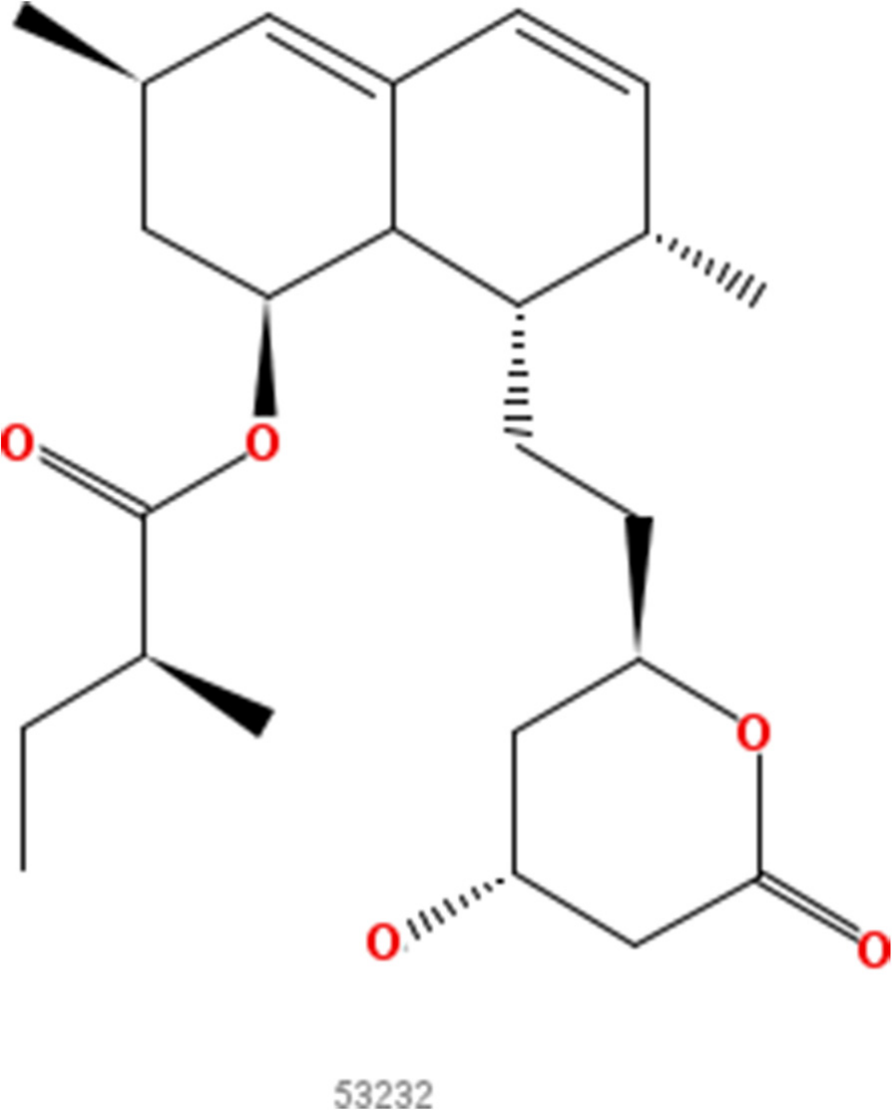
**Lovastatin.** 2D structure depiction of Lovastatin (PubChem CID 53232).

**Figure 5:**
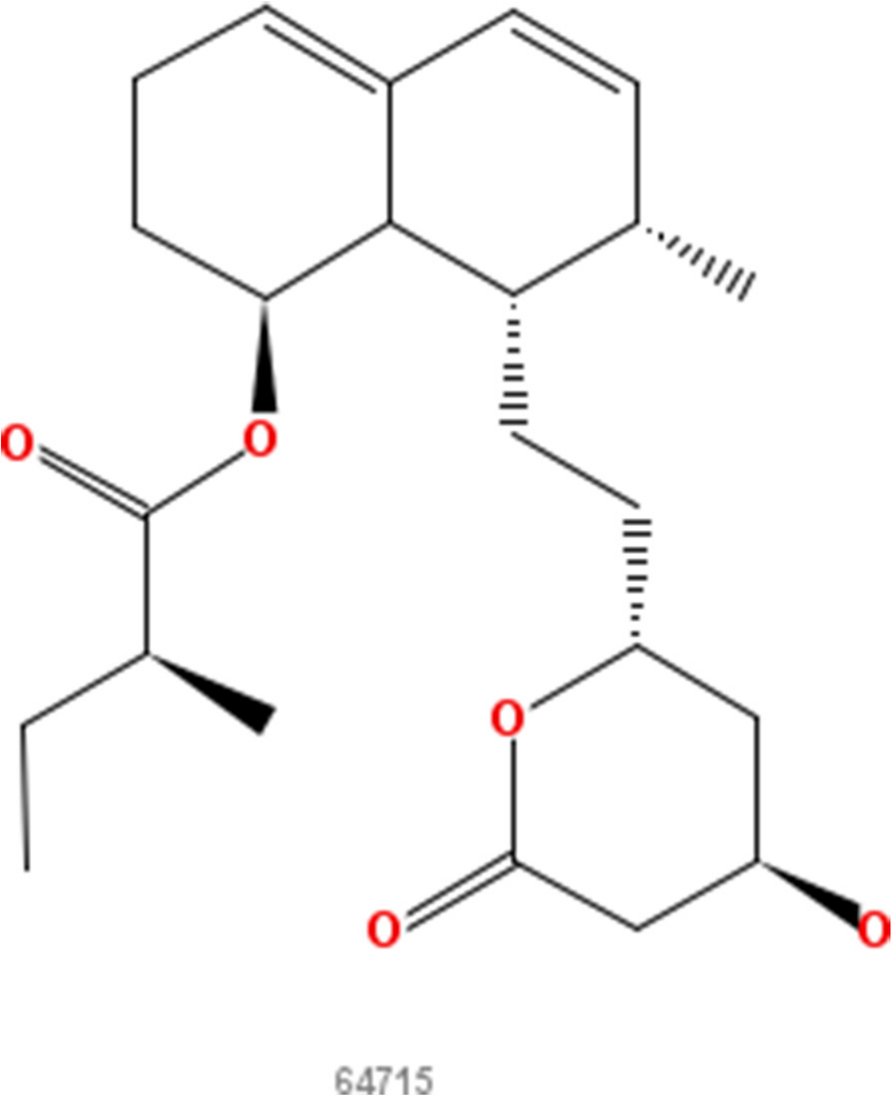
**Mevastatin.** 2D structure depiction of Mevastatin (PubChem CID 64715).

**Figure 6:**
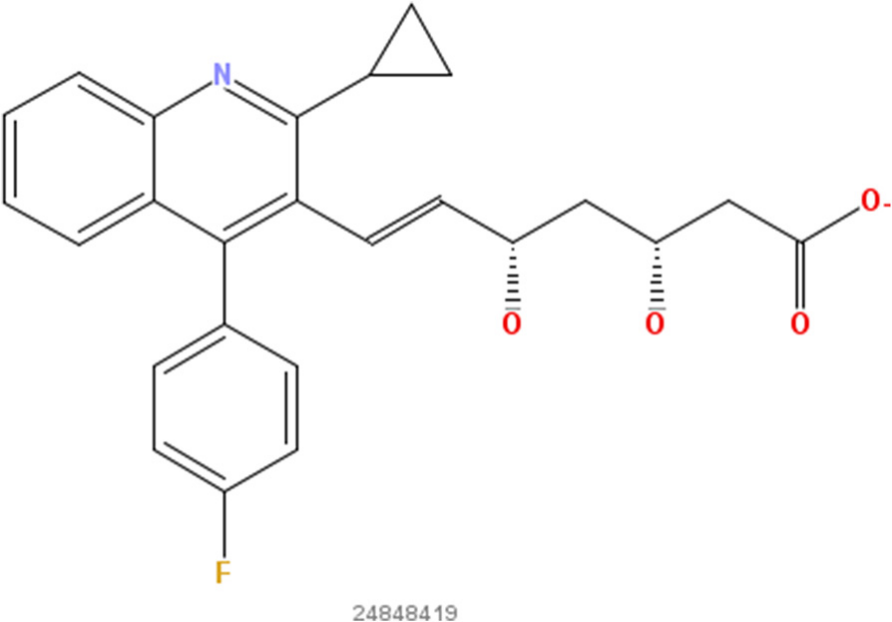
**Pitavastatin.** 2D structure depiction of Pitavastatin (PubChem CID 24848419).

**Figure 7:**
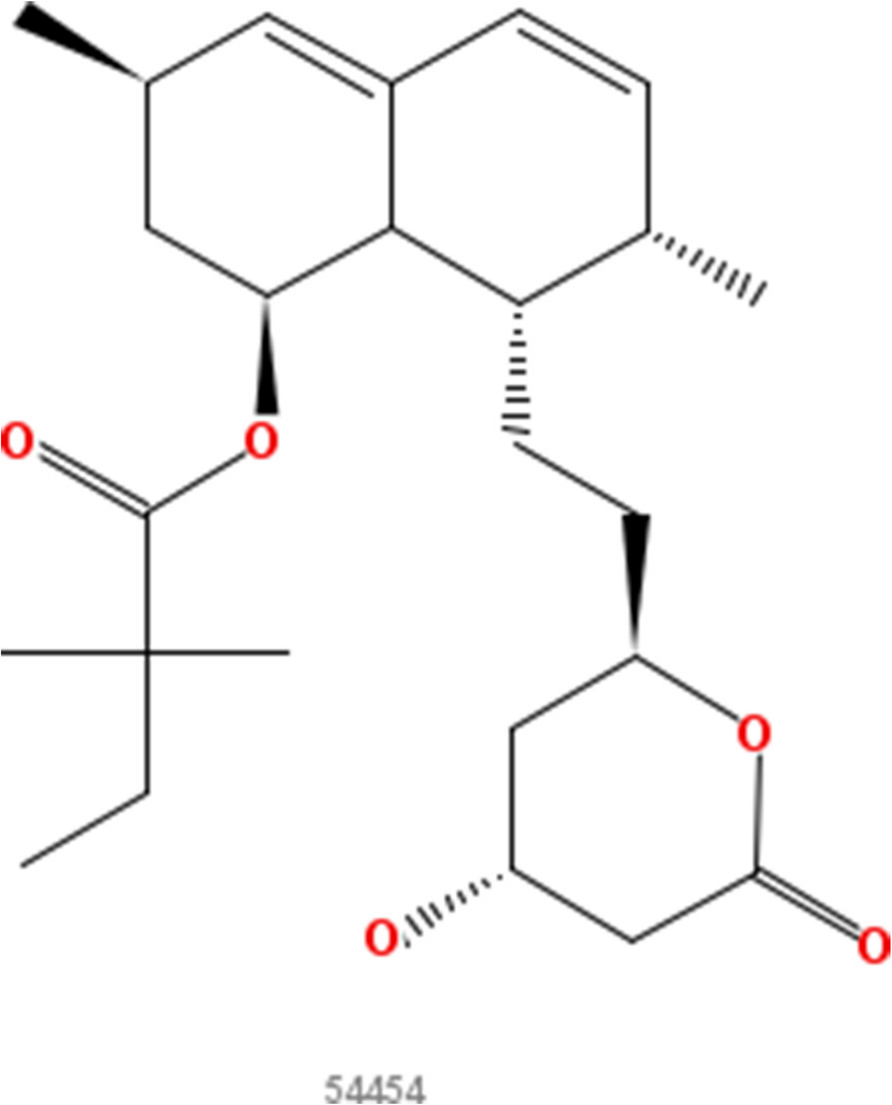
**Simvastatin.** 2D structure depiction of Simvastatin (PubChem CID 54454).

**Figure 8:**
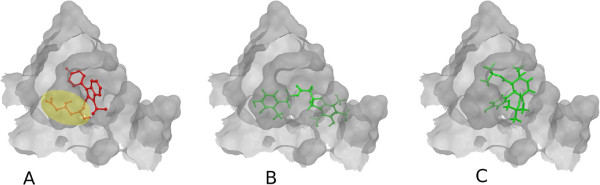
**Selected HMGR ligand binding poses.** Only the active site of the receptor is shown. **A**: Fluvastatin receptor binding. Original position of fluvastatin in the HMGR ([PDB:1HWI]) receptor. The hand-selected important fragment is marked in yellow. **B**: Best docking of best MCS similarity search hit in the HMGR ([PDB:1HWI]) receptor. **C**: Best docking of best MCS _*ext*_ similarity search hit in the HMGR ([PDB:1HWI]) receptor.

In our approach A experiments we first consider the problem of inhibition of the enzyme HMG-CoA reductase (HMGR). Well-known inhibitors of HMGR are chemicals from the drug class of statins (HMG-CoA reductase inhibitors). Most of them are marketed drugs or drugs under development. Inhibition of HMGR lowers the cholesterol levels and prevents cardiovascular diseases [13], which are a major problem in developed countries as coronary artery disease affects 13 to 14 million adults in the United States alone [14]. The statins are structurally quite similar as can be seen in Figures 2, 3, 4, 5, 6 and 7. All of them are competitive inhibitors of HMGR with respect to binding of the substrate HMG-CoA, but not with respect to binding of NADPH [15]. The protein receptor used in the docking procedure is the structure of HMGR co-crystallized with fluvastatin (Figure 8, CID 446155), which is available in the PDB [16] with identifier [PDB:1HWI] [17]. We use two sets of known ligands that are mixed with the decoys and provide the reference compound in this first set of experiments: first the set of statins and second the HMGR ligands provided by the DuD HMGR data set. In case the statins are used as ligand set, we repeat the experiment with each statin as query compound, otherwise we randomly select ten DuD HMGR ligands and use each one of those as query compound.

#### PPAR ***γ***

**Figure 9:**
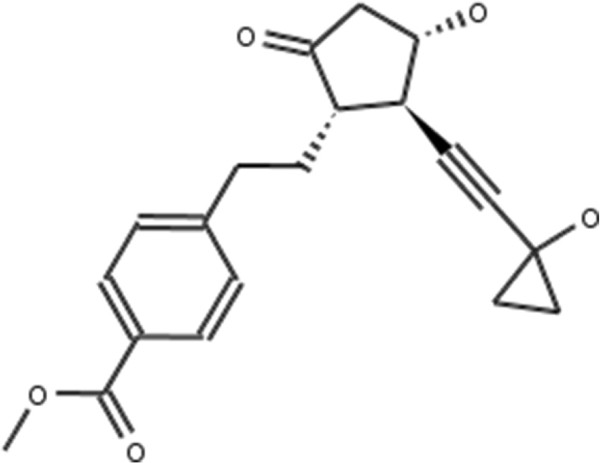
**PPAR*γ* approved active drugs.** Eight DrugBank listed PPAR *γ* active drugs that have “approved” status. The DrugBank ID is shown with the molecule.

In addition to HMGR we test the by-hand selection approach on the PPAR *γ* data set. The PPAR *γ* receptor binds peroxisome proliferators such as hypolipidemic drugs and fatty acids. Once activated by a ligand, the receptor binds to a promoter element in the gene for acyl-CoA oxidase and activates its transcription. It therefore controls the peroxisomal beta-oxidation pathway of fatty acids and is a key regulator of adipocyte differentiation and glucose homeostasis [18]. The DrugBank [9] database lists - amongst others - these eight drugs that are market approved and known PPAR *γ* interactors: Bezafibrate, Glipizide, Ibuprofen, Mesalazine, Sulfasalazine, Balsalazide, Rosiglitazone and Pioglitazone. An overview of the drugs, their DrugBank IDs and structures are given in Table 3 and Figure 9.

**Table 3:** **PPAR** ***γ* market approved drugs**

**DrugBank ID**	**Drug name**
DB01393	Bezafibrate
DB01067	Glipizide
DB01050	Ibuprofen
DB00244	Mesalazine
DB00795	Sulfasalazine
DB01014	Balsalazide
DB00412	Rosiglitazone
DB01132	Pioglitazone

PPAR ***γ*** market approved drugs with DrugBank ID and drug name.

We use the same query and database set-up as with the HMGR experiments.

#### Directory of useful decoys

As database for the approach B experiments, we use the Directory of useful Decoys that is designed to avoid bias in docking and screening studies. The DuD database consists of more than 95,000 decoy structures and 2,950 ligand structures (more than 30 decoy structures per ligand) for 40 protein targets including HMGR. We chose eight target structures from the DuD database in addition to HMGR and PPAR *γ*. The original forty DuD target sets are grouped into six classes: nuclear hormone receptors, kinases, serine proteases, metalloenzymes, folate enzymes and other enzymes. We selected the additional protein targets to cover all six classes: estrogen receptor (ER; antagonists) from the class of nuclear hormone receptors, p38 mitogen-activated protein kinase (P38 MAP) and thymidine kinase (TK) for the class of kinases, factor Xa (FXa) for the class of serine proteases, adenosine deaminase (ADA) for the class of metalloenzymes, dihydrofolate reductase (DHFR) for the class of folate enzymes and the acetylcholine esterase (AChE) as well as cyclooxygenase 2 (COX-2) for the remaining “other enzyme” class. An overview of the DuD data sets used in this study is given in Table 2. For DHFR three and for FXa two ECFP similarities could not be calculated due to software problems (the applied RDKit software was not able to process those molecules). The respective compounds were removed from the experimental setting. For this second set of experiments we randomly select ten of the ligands as reference compounds and mix the remaining ligands with the decoys. This procedure is repeated ten times.

#### ChEMBL activity classes

To strengthen the findings on the mining-based experiments with the DuD data sets we add another set of compound data sets compiled by Li and Bajorath [11]. They selected compounds by activity class from the ChEMBL database (ChEMBL level 9) with restrictions to the reported potency values (at least 10*μM*) and the contained number of distinct Bemis and Murcko scaffolds [19] (at least 3). After evaluation they report 50 activity classes as test cases for benchmark calculations. We use 25 (random selection) of those 50 activity classes (actually 49 – activity class 168 only provides one ligand and is therefore removed) as ligand sets. We randomly select ten ligands per activity class (or all available if the number of compounds in the activity class is smaller than ten). As background database we randomly select a set of 100,000 compounds from the ZINC [12] “All Purchasable” data set. For this set of experiments we randomly select one of the ligands as reference compound and mix the remaining ligands with the decoys. This procedure is repeated ten times.

### Evaluation measures

To evaluate the performance of the similarity measures, we consider the enrichment factor (EF) [20] that is achieved by a virtual screening. The enrichment factor reflects the amount of known related structures in the first *x%* of the ranked database. In practice, often only the highest ranked compounds are of interest and considered further in the drug discovery pipeline. The enrichment factor is defined for certain fractions of the database:

(3) $$\text{EF}(\%)=\frac{\left(N_{\text{active}(\%)}/N_{\left(\%\right)}\right)}{\left(N_{\text{active}}/N_{\text{all}}\right)},$$

where *EF*(*%*) is given for the specified percentage of the ranked database, *N*_*a* *c* *t* *i* *v* *e*(*%*)_ is the number of active compounds in the selected subset of the ranked database, *N*_(*%*)_ is the number of compounds in the subset, *N*_*a* *c* *t* *i* *v* *e*_ is the number of active molecules in the data set and *N*_*a* *l* *l*_ is the number of compounds in the database. For an easier interpretation of the EF values, it is helpful to compare them to the maximum possible enrichment at the specified fraction of the database:

For easier comparison we do not use the EF(%) directly, but the difference of maximum possible enrichment and achieved enrichment:

(4) $$\Delta_{\text{EF}}=EF_{\text{max}}-\text{EF}(\%).$$

Keep in mind that for *Δ*_*E* *F*_ smaller values are better and the optimal *Δ*_*E* *F*_ is zero. In our study, we use the top 1%, 5% and 10% fractions of the ranked database to calculate the EF values. In the results section of this work we restrict ourselves to showing the *Δ*_*E* *F*_ values.

The first step in the docking process was the automatic preparation of the complete PDB structure of [PDB:1HWI] with the Protein Preparation Wizard of the Maestro Suite. Since there are four identical binding sites, the docking was performed with only one of them. At some binding sites ADP is bound nearby. Since ADP does not participate in statin binding [17] the binding site mainly formed by chain D with some contribution of chain C was chosen, which lacks ADP. In order to speed up the docking procedure, the multi-mer was simplified by removing the redundant chains A and B. The receptor preparation was completed by the manual removal of all waters, the ligand molecule and the ADPs of the other binding site formed by chain C and D. The selected drug candidates were prepared using Ligprep 2.5. In a preprocessing step of the docking procedure the receptor grid for the chosen binding site was pre-calculated using the Glide 5.7 Receptor Grid Generation. The co-crystallized fluvastatin in the chosen binding site was used as reference ligand. Subsequently the rigid receptor docking was performed with the extra precision mode of the Glide 5.7 Ligand Docking application.

### Docking procedure

Molecular docking was applied in order to assess if the extensions to the structural similarity measures are suitable for virtual screening. For the HMGR experiments we did the docking ourselves, for the second experiment we used the docking scores as provided in the DuD database. We now describe the docking procedure applied in the by-hand HMGR experiment.

HMGR is a tetra-mer with four identical binding sites whereas two chains contribute residues to one binding site. In the PDB six co-crystallizations of HMGR are available, each with one statin: atorvastatin ([PDB:1HWK]), fluvastatin ([PDB:1HWI]), simvastatin ([PDB:1HW9]), compactin ([PDB:1HW8]), rosuvastatin ([PDB:1HWL]) and cerivastatin ([PDB:[1HWJ]). A comparison of the CoA bound binding sites with the statin bound binding sites showed rearrangements. In the statin bound binding sites some residues are disordered which fold to an *α*-helix when CoA is bound. In the presence of the *α*-helix, a narrow pantothenic acid-binding pocket is formed making it impossible for statins to bind. Instead a hydrophobic groove is formed that accommodates the hydrophobic moieties of the statins which accounts for a tighter binding of the statins [17]. Since we are interested in drug candidates with a similar binding ability as the statins, we focus on the statin bound HMGR structures. According to Istvan *et al.*[17] the orientation of the side chains in the binding sites does not differ among the statins. This was confirmed by a superposition of the six PDB structures with Pymol (http://www.pymol.org/). Due to this we chose to perform a rigid receptor cross-docking of the structural similar drug candidates to 1hwi with Glide 5.7 from the Maestro Suite of Schrödinger. If not indicated otherwise, the default settings were used.

## Results and discussion

### By-hand experiments

**Figure 10:**
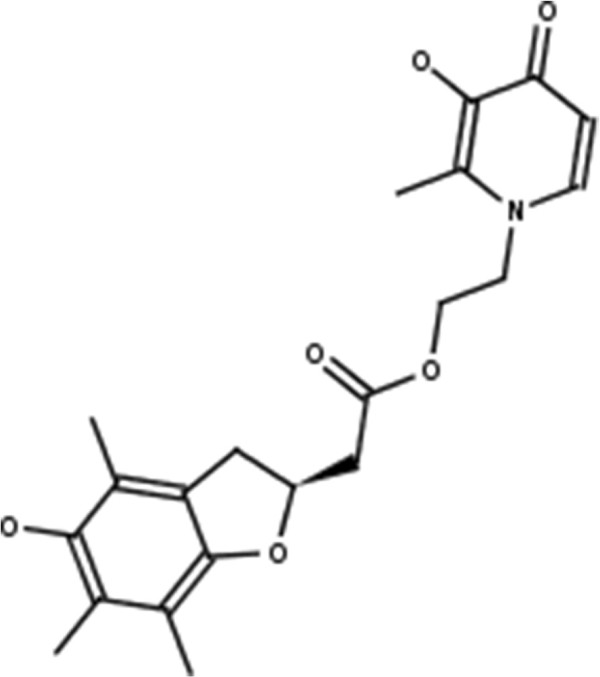
**ZINC26851.** 2D structure depiction of ZINC26851 from the MCS similarity ranking. Rank difference: *Δ*_*Rank*_=−16.

**Figure 11:**
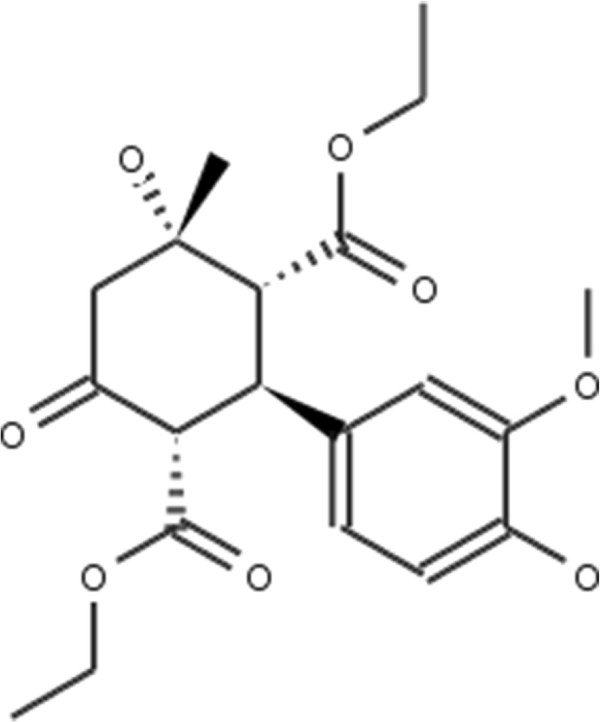
**ZINC588723.** 2D structure depiction of ZINC588723 from the MCS similarity ranking. Rank difference: *Δ*_*Rank*_=6.

**Figure 12:**
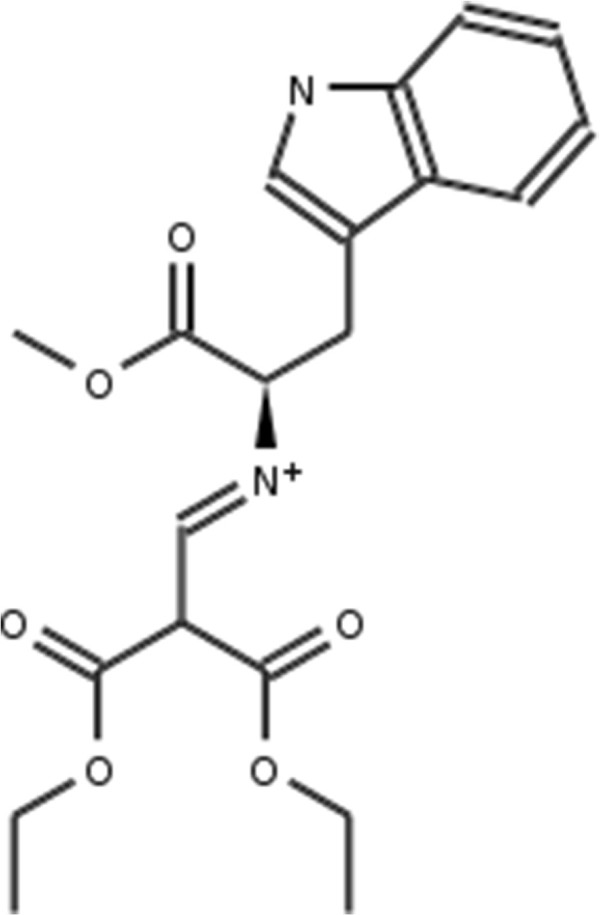
**ZINC714466.** 2D structure depiction of ZINC714466 from the MCS similarity ranking. Rank difference: *Δ*_*Rank*_=0.

**Figure 13:**
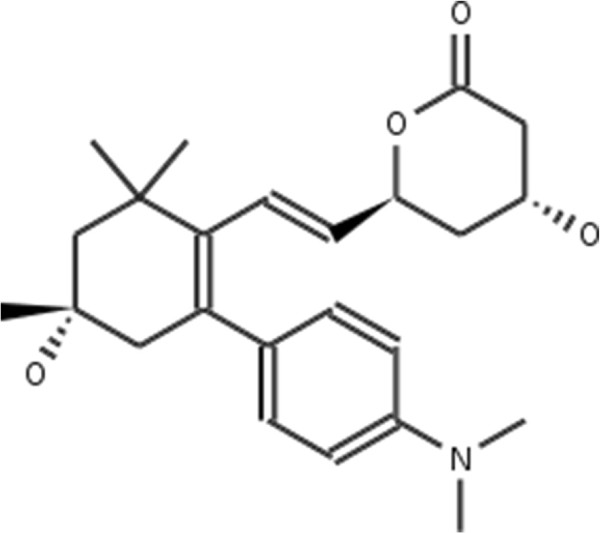
**ZINC4628438.** 2D structure depiction of ZINC4628438 from the MCS similarity ranking. Rank difference: *Δ*_*Rank*_=11.

In the first set of experiments we extract the binding-relevant knowledge used to extend the structural similarity measures by literature review and support the process by MCS similarity ranking and docking calculations. We therefore rank the screening database (including decoys and statin ligands) with respect to fluvastatin using *sim*_*M* *C* *S*_. Subsequently, we docked the top 25 compounds of the similarity ranking to the HMGR receptor. Looking at the docking results in Table 4 (and the long version in the Additional file 1: Table S1), it can be seen that only one compound (CID 60823) has a good docking score. This is atorvastatin, one of the two statins found in the top 25 of the MCS similarity ranking. All other compounds have rather weak docking scores. Four structures from this ranking are shown in Figures 10, 11, 12, 13 and the docking of the best non-statin is shown in Figure 8B. It can clearly be seen that the highlighted part of the structure of fluvastatin (Figure 3 and Figure 8A) or something structurally similar, is not present in any of the structures (non-statins). According to Istvan *et al.*[17], this part mimics the original binding ligand and consequently is essential for binding. The hydrophobic part of the statins is responsible for the nano-molar affinity of the statins but not sufficient for inhibitory binding on its own. Taking those facts into consideration, we decided to use the highlighted hydrophilic part of fluvastatin as background knowledge in our study. As described in the Materials and methods Section, the substructure was fragmented and used to derive a binary occurrence fingerprint of length 57 for the extended similarity measure (1).

**Table 4:** Results of the docking run (MCS top 25)

**Rank**	**CID**	**Score**	***R*** ***a*** ***n*** ***k***_***M*** ***C*** ***S***_	$\Delta_{\text{Ran}k_{\text{MCS}}}$
1	60823	-10.564	2	-1
2	ZINC02336737	-5.808526	13	-11
3	ZINC00026851	-5.699634	19	-16
4	ZINC00588719	-5.568737	11	-7
5	ZINC00599752	-5.46502	5	0
6	ZINC00588053	-5.463745	16	-10
7	ZINC00864379	-5.291673	15	-8
8	ZINC01253780	-5.211104	14	-6
9	ZINC00714466	-5.149133	9	0
10	ZINC00588723	-5.14689	4	6

Results of the docking run (MCS top 25). ***Δ***_*Rank*_=*Rank*_*docking*_−*Rank*_*MCS*_. A negative ***Δ***_*Rank*_ value means, in the MCS similarity the compound is ranked lower, a positive ***Δ***_*Rank*_ that it is ranked higher than by the docking procedure. For the complete table, refer to Additional file 1: Table S1.

**Figure 14:**
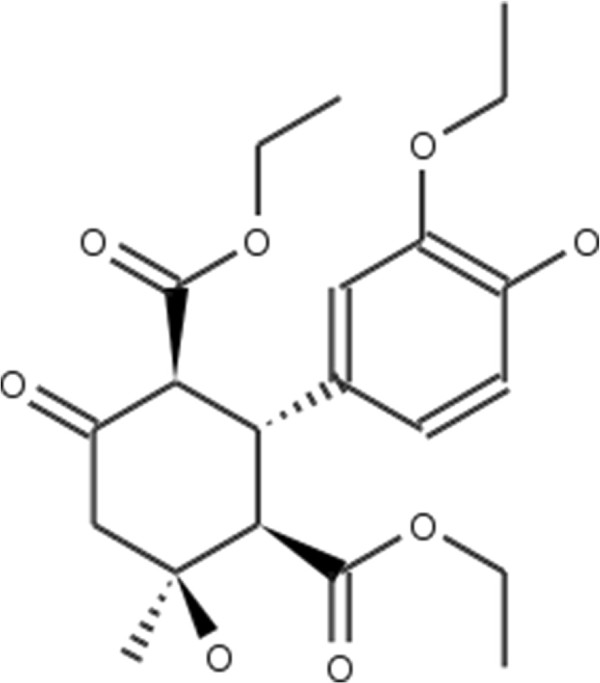
**ZINC599752.** 2D structure depiction of ZINC599752 from the extended MCS similarity ranking (MCS _*ext*_). Rank difference: *Δ*_*Rank*_=11.

**Figure 15:**
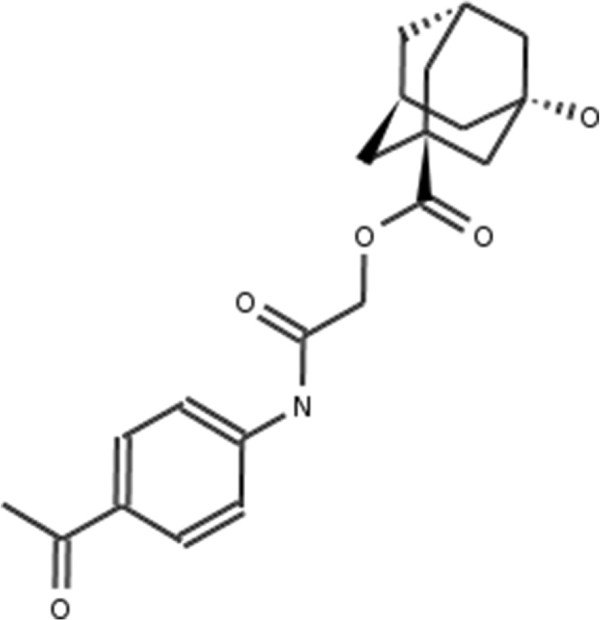
**ZINC1112466.** 2D structure depiction of ZINC1112466 from the extended MCS similarity ranking (MCS _*ext*_). Rank difference: *Δ*_*Rank*_=2.

**Figure 16:**
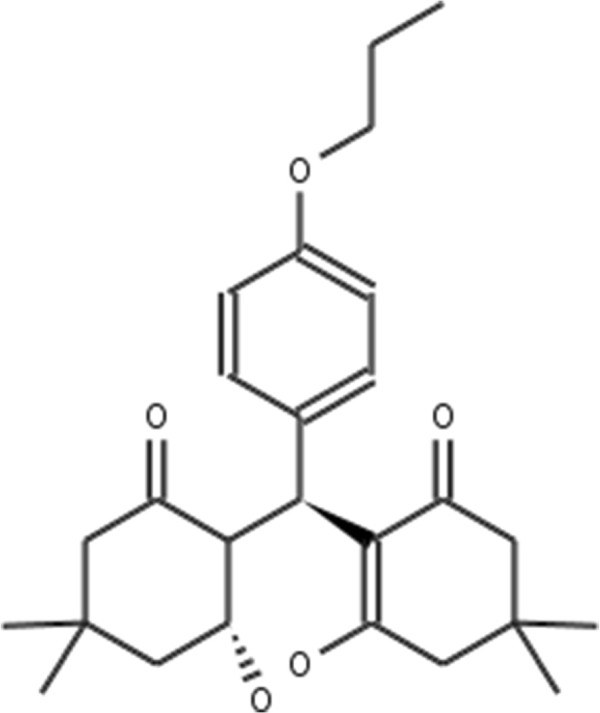
**ZINC4597014.** 2D structure depiction of ZINC4597014 from the extended MCS similarity ranking (MCS _*ext*_). Rank difference: *Δ*_*Rank*_=−12.

**Figure 17:**
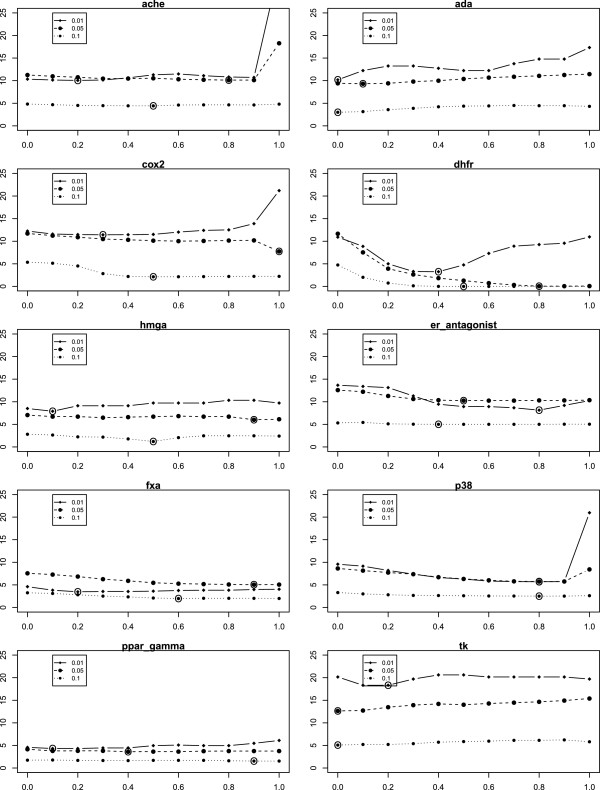
**ZINC19313623.** 2D structure depiction of ZINC19313623 from the extended MCS similarity ranking (MCS _*ext*_). Rank difference: *Δ*_*Rank*_=−2.

We then calculated a similarity ranking with the extended MCS similarity measure and again docked the top 25 compounds. The results of docking the top 25 compounds of the extended MCS similarity ranking are shown in Table 5 (see Additional file 1: Table S2 of the supplement). Four structures from the ranking are shown in Figures 14, 15, 16 and 17. The docking scores are clearly improved in comparison to those of the structures found by the MCS similarity ranking given in Table 4 (see Additional file 1: Table S1). This means that the compounds found will very likely have a higher binding affinity to the receptor. Figures 8, 9 and 10 show the original position of fluvastatin and dockings of the two non-statins with the best docking score from the two similarity rankings. It can be seen that the ligand of the extended MCS similarity (in Figure 10) enters the active site much better than the one of the MCS similarity (in Figure 9).

**Table 5:** **Results of the docking run (MCS**_***ext***_** top 25)**

**Rank**	**CID**	**Score**	${\text{Rank}}_{{\text{MCS}}_{\text{ext}}}$	$\Delta_{{\text{Rank}}_{\text{MCSext}}}$
1	ZINC00588723	-10.382184	16	-15
2	24848419	-7.980885	3	-1
3	ZINC01253780	-7.385909	9	-6
4	ZINC00625939	-7.157018	11	-7
5	ZINC01032240	-7.104563	5	0
6	ZINC00864379	-7.052449	10	-4
7	ZINC00026851	-6.910078	19	-12
8	ZINC00714466	-6.702119	6	2
9	ZINC01112466	-6.667553	7	2
10	64715	-6.654007	12	-2

Results of the docking run (MCS_*ext*_ top 25). ***Δ***_*Rank*_=*Rank*_*docking*_−*Rank*_*MCS or ECFP*_. A negative ***Δ***_*Rank*_ value means, in the extended similarity the compound is ranked lower, a positive ***Δ***_*Rank*_ that it is ranked higher than by the docking procedure. For the complete table, refer to Additional file 1: Table S1.

As last experiment for the by-hand approach, we calculated similarity rankings with the ECFP similarity and also with an extended version of the ECFP similarity. We use the same binding-relevant substructure as for the MCS similarity. Comparing the differences in enrichment factors of the ligand structures in the ranked databases (MCS and ECFP similarity rankings) with the respective extended variants (see Table 6), it is clear that the extension is beneficial in all cases. Especially the MCS similarity, that shows a slightly weaker performance than the ECFP similarity, benefits from the similarity extension. Here an improvement of *Δ*_*E* *F*_ can be seen in all except one cases (if further improvement is possible). For ECFP a decrease in *Δ*_*E* *F*_ can be seen in all except four cases.

**Table 6:** ***Δ***_***EF***_** values for HMGR**

**Query vs. DB**	**MCS**			**MCS **_***ext***_		
	**1%**	**5%**	**10%**	**1%**	**5%**	**10%**
stat vs DuD_*all*_	63.5 ± 14.1	10.1 ± 3.7	4.5 ± 2.3	**16.7** ± 18.3	**0.0** ± 0.0	**0.0** ± 0.0
stat vs DuD_*set*_	61.2 ± 13.6	9.4 ± 4.4	3.1 ± 2.2	**28.9** ± 26.1	**1.7** ± 1.8	**0.0** ± 0.0
lig vs DuD_*all*_	14.1 ± 1.7	1.1 ± 0.3	0.0 ± 0.1	**3.1** ± 0.7	**0.3** ± 0.1	0.0 ± 0.0
lig vs DuD_*set*_	0.0 ± 0.0	2.1 ± 0.5	0.0 ± 0.0	0.0 ± 0.0	**1.7** ± 0.2	0.0 ± 0.0
	**ECFP**			**ECFP**_***ext***_		
	**1%**	**5%**	**10%**	**1%**	**5%**	**10%**
stat vs DuD_*all*_	50.0 ± 0.0	10.0 ± 0.0	5.0 ± 0.0	**8.3** ± 20.4	**1.7** ± 0.0	**0.0** ± 0.0
stat vs DuD_*set*_	52.0 ± 0.0	10.1 ± 0.0	5.0 ± 0.0	**20.2** ± 20.2	**0.0** ± 0.0	**0.0** ± 0.0
lig vs DuD_*all*_	6.1 ± 1.2	1.4 ± 0.2	0.1 ± 0.1	**5.9** ± 1.5	1.4 ± 0.3	**0.0** ± 0.0
lig vs DuD_*set*_	0.1 ± 0.1	0.9 ± 0.2	0.0 ± 0.0	**0.0** ± 0.0	**0.4** ± 0.1	0.0 ± 0.0

***Δ***_*EF*_ value for all four similarity methods for the hand-selection experiments with HMGR at 1%, 5% and 10% of the database. Improvements compared to the non-extended variant are marked in bold print. stat = statines.

**Figure 18:**
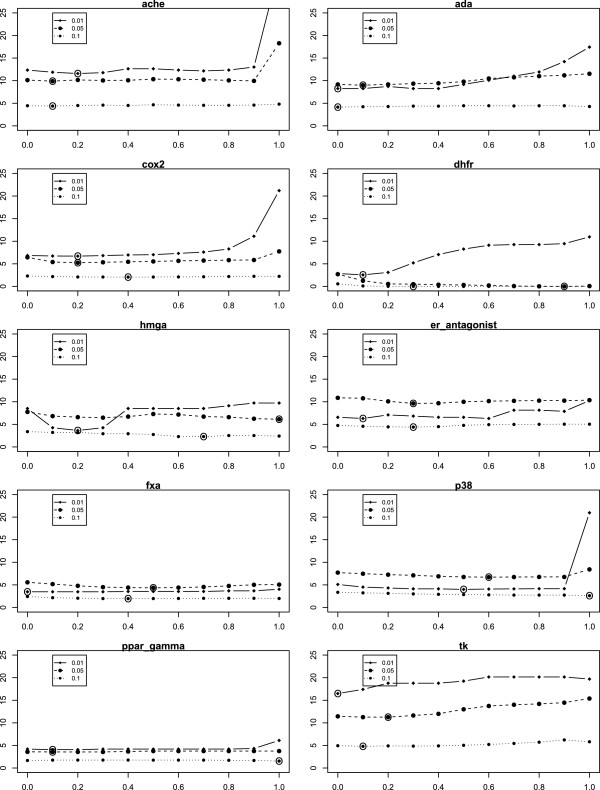
**PPAR*γ* binding relevant substructures.** Binding relevant substructures used for calculating the bind_fp fingerprint for the PPAR *γ* by-hand experiments (approach A).

For the second data set we use for testing the by-hand approach, PPAR *γ*, we shorten the selection procedure. By visual inspection of the eight approved drugs shown in Table 3 and Figure 9 as well as binding information on Rosiglitazone given in by Liberato *et al.*[21] we select two binding relevant substructures as shown in Figure 18. As described in the Materials and methods Section, the substructures were fragmented and used to derive a binary occurrence fingerprint for the extended similarity measure (1). The results for the similarity rankings that are calculated in analogy to the HMGR by-hand experiments are given in Table 7. The results clearly show that the reduced effort to extract the binding-relevant information has direct impact on the ranking performance. Only in half of the settings (MCS lig vs. DUD_*set*_, ECFP lig vs. DUD_*all*_ and ECFP lig vs. DUD_*set*_) we see improvements of the extended similarity measures in comparison the base similarity measures. From that we conclude that it is of high importance to be very careful on selecting the binding-relevant structural information when using the presented approach A (by-hand selection).

**Table 7:** ***Δ***_***EF***_** values for PPAR** ***γ***

**Query vs. DB**	**MCS**			**MCS**_***ext***_		
	**1%**	**5%**	**10%**	**1%**	**5%**	**10%**
ChBa vs DuD_*all*_	82.9 ± 6.5	15.0 ± 1.3	7.0 ± 1.1	**79.7** ± 6.5	15.0 ± 1.9	7.3 ± 0.8
ChBa vs DuD_*set*_	73.8 ± 10.5	12.2 ± 3.4	5.2 ± 1.6	80.0 ± 6.5	14.4 ± 1.8	7.0 ± 0.9
lig vs DuD_*all*_	10.3 ± 5.7	7.9 ± 3.3	4.1 ± 2.7	11.0 ± 6.1	8.2 ± 7.5	**2.9** ± 2.8
lig vs DuD_*set*_	8.2 ± 3.6	6.9 ± 2.8	3.0 ± 2.3	**8.0** ± 6.1	7.0 ± 9.5	**1.9** ± 2.6
	**ECFP**			**ECFP**_***ext***_		
	**1%**	**5%**	**10%**	**1%**	**5%**	**10%**
ChBa vs DuD_*all*_	79.7 ± 9.3	13.8 ± 1.9	6.6 ± 0.6	**78.1** ± 8.8	14.7 ± 1.6	7.0 ± 0.9
ChBa vs DuD_*set*_	70.7 ± 11.5	12.9 ± 1.6	5.5 ± 1.9	78.5 ± 8.9	14.1 ± 2.3	6.7 ± 1.1
lig vs DuD_*all*_	6.9 ± 3.3	7.4 ± 11.1	2.4 ± 2.6	10.0 ± 8.3	**4.6** ± 1.1	**1.2** ± 1.2
lig vs DuD_*set*_	4.2 ± 2.1	6.1 ± 1.3	0.9 ± 1.1	**3.9** ± 8.7	**3.5** ± 1.9	0.9 ± 0.7

***Δ***_*EF*_ value for all four similarity methods for the hand-selection experiments with PPAR ***γ*** at 1%, 5% and 10% of the database. Improvements compared to the non-extended variant are marked in bold print. ChBa = ChemBank ligands.

### Mining-based experiments

In the following, we first assess for both data-mining based variants (B1: all known ligands used to calculate the fragment occurrence fingerprint or B2: only part of them used), if the extension of the MCS and the ECFP similarity measures with the data mining derived fingerprint improves the quality of the similarity ranking. Second we compare the data mining approach with the by-hand approach for the HMGR data set. The results for variant B1 are given in Tables 8, 9 and 10. To see how the data mining based approach performs, when only few ligand structures are available as background knowledge, we re-ran the experiments with variant B2: using only ten per cent randomly chosen from the respective DuD ligand sets (20% due to smaller ligand set sizes in case of the HMGR, ADA and TK data sets) to extract background knowledge. The results using variant B2 are given in Tables 11, 12 and 13.

**Table 8:** **Mean** ***Δ***_***EF***_** and standard deviation for the MCS and MCS**_***ext***_** similarity methods (approach B1)**

**DuD set**	**MCS**			**MCS**_***ext***_		
	**1%**	**5%**	**10%**	**1%**	**5%**	**10%**
HMGR	8.5 ± 4.5	7.0 ± 6.8	2.8 ± 3.6	**4.6** ± 9.8	**2.0** ± 1.0	**0.5** ± 0.3
ER	13.6 ± 7.6	12.6 ± 4.1	5.3 ± 1.7	**13.1** ± 7.0	**11.4** ± 2.8	**3.7** ± 0.9
PPAR *γ*	4.6 ± 10.6	1.2 ± 5.4	1.7 ± 2.8	**4.6** ± 11.0	3.8 ± 5.5	**1.5** ± 2.9
P38 MAP	9.6 ± 7.9	8.6 ± 3.7	3.3 ± 1.8	**3.8** ± 5.4	**4.8** ± 4.2	**2.4** ± 2.1
TK	20.1 ± 4.4	12.6 ± 2.1	5.1 ± 1.6	**18.3** ± 5.3	**12.3** ± 2.7	**4.0** ± 1.3
FXa	4.6 ± 11.2	7.6 ± 3.8	3.3 ± 1.8	**3.5** ± 11.0	**6.4** ± 4.6	**2.5** ± 2.5
ADA	10.1 ± 6.4	8.2 ± 3.0	4.3 ± 3.6	**9.2** ± 4.8	**7.7** ± 2.0	**2.3** ± 0.8
DHFR	10.9 ± 10.6	11.7 ± 2.9	4.7 ± 1.1	**3.1** ± 5.0	**0.3** ± 0.3	**0.0** ± 0.0
AChE	10.3 ± 12.5	11.3 ± 4.7	4.8 ± 2.5	**10.0** ± 11.8	**9.5** ± 5.8	**4.4** ± 3.0
COX-2	12.3 ± 9.2	11.7 ± 2.2	5.3 ± 1.1	**10.7** ± 10.3	**10.1** ± 3.8	**2.2** ± 2.6
w/d/l				**10** / 0 / 0	**9** / 0 / 1	**10** / 0 / 0

Mean ***Δ***_*EF*_ and standard deviation for the MCS and MCS_*ext*_ similarity methods at 1%, 5% and 10% of the database (receptor specific decoy set DuD_*set*_). The extension fingerprint is calculated from all ligands (approach B1). Improvements of MCS_*ext*_ compared to MCS are marked with bold print. w/d/l = wins/draws/losses.

**Table 9:** **Mean** ***Δ***_***EF***_** and standard deviation for the ECFP and ECFP**_***ext***_** similarity methods (approach B1)**

**DuD set**	**ECFP**			**ECFP**_***ext***_		
	**1%**	**5%**	**10%**	**1%**	**5%**	**10%**
HMGR	8.7 ± 9.4	6.8 ± 8.5	4.2 ± 5.6	**0.0** ± 0.0	**2.8** ± 2.2	**0.9** ± 0.5
ER	8.0 ± 4.0	7.4 ± 3.9	6.7 ± 4.6	**6.3** ± 4.8	9.2 ± 1.6	**3.3** ± 1.2
PPAR *γ*	1.3 ± 0.7	7.1 ± 11.2	1.0 ± 0.7	4.2 ± 11.1	**3.6** ± 5.7	1.8 ± 2.8
P38 MAP	7.0 ± 5.9	5.9 ± 3.0	3.4 ± 2.0	**2.8** ± 5.7	**5.0** ± 4.1	**2.4** ± 2.1
TK	9.8 ± 6.0	12.1 ± 8.9	10.9 ± 6.4	16.5 ± 8.4	**11.4** ± 3.6	**4.3** ± 2.0
FXa	7.4 ± 11.3	2.4 ± 2.0	1.7 ± 1.5	**3.5** ± 11.0	4.3 ± 5.3	2.0 ± 2.7
ADA	6.3 ± 3.3	6.4 ± 4.5	8.9 ± 6.0	8.3 ± 7.1	7.7 ± 2.0	**2.4** ± 1.0
DHFR	2.5 ± 2.0	1.8 ± 1.5	1.8 ± 1.5	**1.9** ± 0.9	**0.1** ± 0.1	**0.0** ± 0.0
AChE	15.0 ± 11.2	5.2 ± 2.3	6.8 ± 3.8	**11.0** ± 12.0	9.4 ± 5.6	**4.0** ± 2.8
COX-2	8.7 ± 10.6	3.4 ± 1.9	3.4 ± 2.5	**6.7** ± 10.0	5.5 ± 5.0	**2.1** ± 2.6
w/d/l				**7** / 0 / 3	**5** / 0 / 5	**8** / 0 / 2

Mean ***Δ***_*EF*_ and standard deviation for the ECFP and ECFP_*ext*_ similarity methods at 1%, 5% and 10% of the database (receptor specific decoy set DuD_*set*_). The extension fingerprint is calculated from all ligands (approach B1). Improvements of ECFP_*ext*_ compared to ECFP are marked with bold print. w/d/l = wins/draws/losses.

**Table 10:** **Mean** ***Δ***_***EF***_** and standard deviation for the bind_fp similarity method (approach B1)**

**DuD set**	**Bind_fp**		
	**1%**	**5%**	**10%**
HMGA	6.7 ± 1.5 ^∙^°	1.1 ± 0.0 ^∙^°	0.6 ± 0.0 ^∙^°
ER	62.3 ± 15.2	8.7 ± 4.1°	2.7 ± 1.6 ^∙^°
PPAR *γ*	13.2 ± 30.1	2.4 ± 6.1	1.2 ± 3.0°
P38 MAP	24.2 ± 22.2	4.8 ± 4.3°	2.4 ± 2.1 ^∙^°
TK	42.8 ± 24.3	0.9 ± 1.5 ^∙^°	0.0 ± 0.0 ^∙^°
Fxa	21.2 ± 26.9	3.8 ± 5.5°	1.8 ± 2.7°
ADA	26.1 ± 0.0	1.7 ± 0.0 ^∙^°	0.5 ± 0.1 ^∙^°
DHFR	0.0 ± 0.0 ^∙^°	0.0 ± 0.0 ^∙^°	0.0 ± 0.0 ^∙^°
AchE	47.4 ± 34.8	7.7 ± 6.0°	3.8 ± 3.0 ^∙^°
COX-2	71.6 ± 22.6	10.2 ± 6.3°	2.2 ± 2.6 ^∙^°

Mean ***Δ***_*EF*_ and standard deviation for the bind_fp similarity method at 1%, 5% and 10% of the database (receptor specific decoy set DuD_*set*_). The extension fingerprint is calculated from all ligands (approach B1). Cases where bind_fp is better than ECFP or MCS are marked with a ^∙^ or ***°***, respectively.

**Table 11:** **Mean** ***Δ***_***EF***_values and standard deviations for the MCS and MCS_***ext***_** similarity methods (approach B2)**

**DuD set**	**MCS**			**MCS**_***ext***_		
	**1%**	**5%**	**10%**	**1%**	**5%**	**10%**
HMGR	8.5 ± 4.5	7.0 ± 6.8	2.8 ± 3.6	9.1 ± 2.5	**6.5** ± 6.1	**2.0** ± 2.8
ER	13.6 ± 7.6	12.6 ± 4.1	5.3 ± 1.7	**10.2** ± 6.5	**10.6** ± 2.5	**5.0** ± 1.0
PPAR *γ*	4.6 ± 10.6	1.2 ± 5.4	1.7 ± 2.8	**4.5** ± 11.0	3.8 ± 5.5	**1.6** ± 2.9
P38 MAP	9.6 ± 7.9	8.6 ± 3.7	3.3 ± 1.8	**7.1** ± 6.8	**7.3** ± 3.7	**2.7** ± 2.0
TK	20.1 ± 4.4	12.6 ± 2.1	5.1 ± 1.6	**19.7** ± 5.3	14.0 ± 2.1	5.5 ± 1.5
FXa	4.6 ± 11.2	7.6 ± 3.8	3.3 ± 1.8	**3.5** ± 11.2	**6.2** ± 4.7	**2.5** ± 2.6
ADA	10.1 ± 6.4	8.2 ± 3.0	4.3 ± 3.6	12.8 ± 6.4	8.8 ± 3.3	6.1 ± 4.6
DHFR	10.9 ± 10.6	11.7 ± 2.9	4.7 ± 1.1	**3.4** ± 7.0	**2.4** ± 2.1	**0.1** ± 0.1
AChE	10.3 ± 12.5	11.3 ± 4.7	4.8 ± 2.5	**10.1** ± 11.9	**10.4** ± 5.1	**4.4** ± 3.0
COX-2	12.3 ± 9.2	11.7 ± 2.2	5.3 ± 1.1	**11.4** ± 10.3	**10.5** ± 3.7	**2.5** ± 2.5
w/d/l				**8** / 0 / 2	**7** / 0 / 3	**8** / 0 / 2

Mean ***Δ***_*EF*_ and standard deviations for the MCS and MCS_*ext*_ similarity methods at 1%, 5% and 10% of the database (receptor specific decoy set DuD_*set*_). The extension fingerprint is calculated from 10% (20% for HMGR, TK and ADA) of the ligands (approach B2). Improvements of MCS_*ext*_ compared to MCS are marked with bold print. w/d/l = wins/draws/losses.

**Table 12:** **Mean** ***Δ***_***EF***_** values and standard deviations for the ECFP and ECFP**_***ext***_** similarity methods (approach B2)**

	**ECFP**			**ECFP**_***ext***_		
**DuD set**	**1%**	**5%**	**10%**	**1%**	**5%**	**10%**
HMGR	8.7 ± 9.4	6.8 ± 8.5	4.2 ± 5.6	**4.3** ± 9.5	**6.5** ± 4.8	**2.9** ± 2.6
ER	8.0 ± 4.0	7.4 ± 3.9	6.7 ± 4.6	**6.8** ± 7.8	9.6 ± 2.5	**4.4** ± 1.4
PPAR *γ*	1.3 ± 0.7	7.1 ± 11.2	1.0 ± 0.7	4.2 ± 11.0	**3.6** ± 5.6	1.8 ± 2.8
P38 MAP	7.0 ± 5.9	5.9 ± 3.0	3.4 ± 2.0	**4.0** ± 6.0	7.0 ± 3.6	**3.0** ± 1.9
TK	9.8 ± 6.0	12.1 ± 8.9	10.9 ± 6.4	18.8 ± 7.3	**11.8** ± 3.8	**4.9** ± 2.0
FXa	7.4 ± 11.3	2.4 ± 2.0	1.7 ± 1.5	**3.5** ± 11.2	4.5 ± 5.3	2.0 ± 2.7
ADA	6.3 ± 3.3	6.4 ± 4.5	8.9 ± 6.0	8.3 ± 7.1	9.3 ± 2.0	**4.4** ± 1.1
DHFR	2.5 ± 2.0	1.8 ± 1.5	1.8 ± 1.5	5.7 ± 5.5	**0.5** ± 0.8	**0.0** ± 0.0
AChE	15.0 ± 11.2	5.2 ± 2.3	6.8 ± 3.8	**12.2** ± 12.7	10.0 ± 5.4	**4.6** ±2.9
COX-2	8.7 ± 10.6	3.4 ± 1.9	3.4 ± 2.5	**6.8** ± 10.2	5.4 ± 4.9	**2.0** ± 2.7
w/d/l				**6** / 0 / 4	**4** / 0 / 6	**8** / 0 / 2

Mean ***Δ***_*EF*_ and standard deviations for the ECFP and ECFP_*ext*_ similarity methods at 1%, 5% and 10% of the database (receptor specific decoy set DuD_*set*_). The extension fingerprint is calculated from 10% (20% for HMGR, TK and ADA) of the ligands (approach B2). Improvements of ECFP_*ext*_ compared to ECFP are marked with bold print. w/d/l = wins/draws/losses.

**Table 13:** **Mean** ***Δ***_***EF***_** and standard deviation for the bind_fp similarity method (approach B2)**

	**Bind_fp**		
**DuD set**	**1%**	**5%**	**10%**
HMGR	36.5 ± 2.0	20.2 ± 3.2	10.0 ± 2.2
ER	36.8 ± 11.2	20.2 ± 6.0	10.0 ± 3.9
PPAR *γ*	34.3 ± 5.4	19.6 ± 4.1	6.8 ± 2.3
P38 MAP	20.0 ± 0.0	17.3 ± 6.8	8.6 ± 1.4
TK	36.6 ± 13.7	20.2 ± 12.0	10.1 ± 4.2 ^∙^
FXa	9.9 ± 7.6	8.5 ± 0.0	4.2 ± 0.8
ADA	36.7 ± 8.5	20.1 ± 0.0	10.0 ± 0.9
DHFR	36.5 ± 16.8	20.0 ± 7.9	10.0 ± 1.0
AChE	36.4 ± 6.9	20.1 ± 9.2	10.0 ± 3.2
COX-2	35.7 ± 7.5	19.8 ± 6.2	9.9 ± 1.0

Mean ***Δ***_*EF*_ and standard deviation for the bind_fp similarity method at 1%, 5% and 10% of the database (receptor specific decoy set DuD_*set*_). The extension fingerprint is calculated from 10% (20% for HMGR, TK and ADA) of the ligands (approach B2). Cases where bind_fp is better than ECFP or MCS are marked with a ^∙^ or ***°***, respectively.

Testing for the improvement of the extended similarity compared to the baseline similarity, on average, for a given data set, we find the following numbers of wins and losses for a fixed *α* coefficient of 0.3 weighting the contribution of the extension of the similarity measure in Table 11 (MCS vs MCS_*ext*_, approach B2): 8:2 (at 1%), 7:3 (at 5%), 8:2 (at 10%). Similar or even stronger results can be found for other settings, in particular for retrieving 10% of the compounds: 8:2 on Table 12 (ECFP vs. ECFP_*ext*_, approach B2), 10:0 on Table 8 (MCS vs. MCS_*ext*_, approach B1) and 8:2 on Table 9 (ECFP vs. ECFP_*ext*_, approach B1).

Checking whether these results are statistically significant, we chose one of the weakest significance tests, the sign test [22], which is based on only one weak assumption, namely the independence of the measurements. The sign test has a *p*-value ≤0.109 for a result of 8 wins vs. 2 losses, a *p*-value ≤0.0215 for 9 wins vs. 1 loss, and even smaller for 10 wins vs. 0 losses. We apply the sign test to determine whether *Δ*_*E* *F*_ is on average greater for one method compared to another for a given data set.

**Figure 19:**
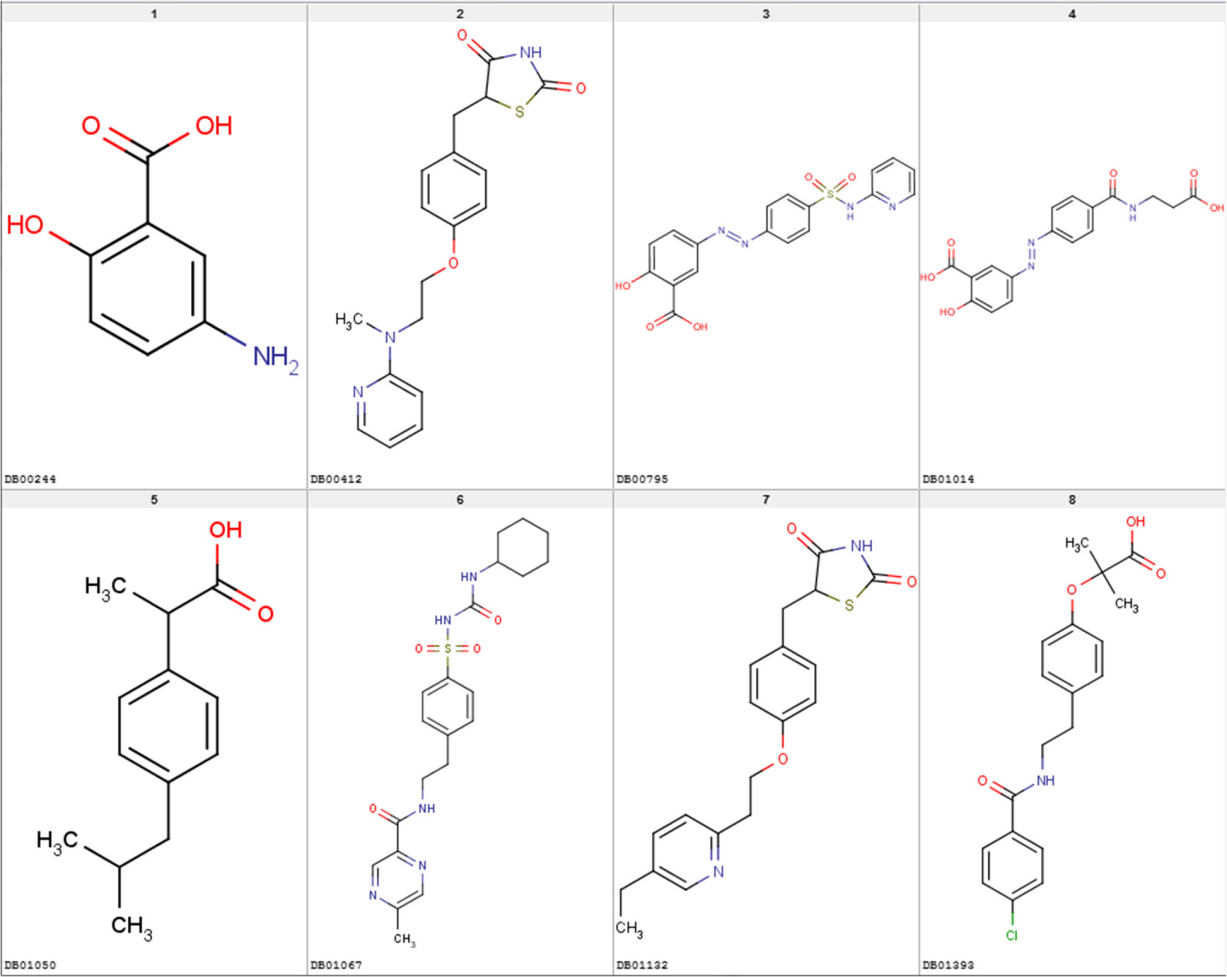
**Plot of*α* vs. Mean*Δ***_***EF***_** for MCS **_***ext***_**.** On the x-axis the values of the combining factor *α* is plottet versus the mean *Δ*_*EF*_ for MCS _*ext*_ on the y-axis. (approach B2).

**Figure 20:**
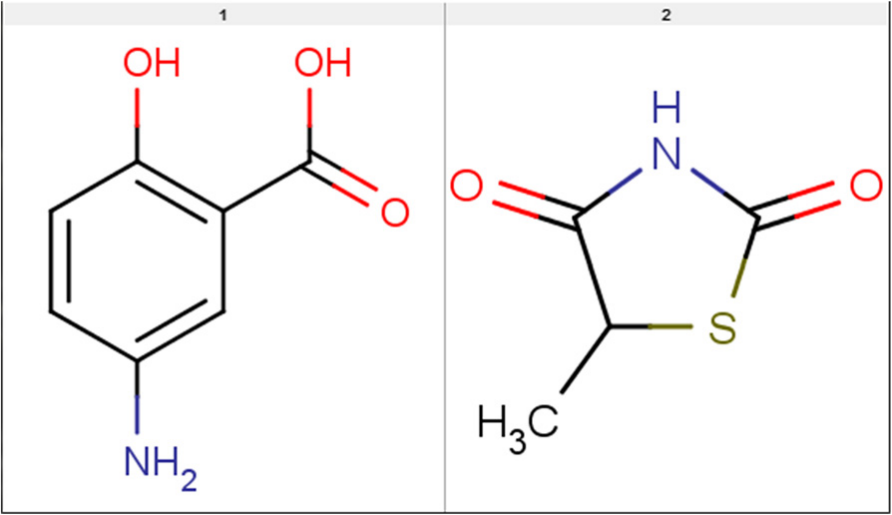
**Plot of*α* vs. Mean*Δ***_***EF***_** for ECFP **_***ext***_**.** On the x-axis the values of the combining factor *α* is plottet versus the mean *Δ*_*EF*_ for MCS _*ext*_ on the y-axis. (approach B2).

While the results already show improvements of the score for a fixed *α* of 0.3, one might be interested in the results for an optimal *α*, which we do not know beforehand. Also, it is interesting to know into which range optimal *α*s fall and whether 0.3 is a suitable default value. Results are shown in Tables 14, 15 and 16 as well as in Figures 19 and 20. As it turns out, the statistics of the number of wins and losses can still be improved, e.g., from 8:2, 7:3, 8:2 to 10:0, 9:0, 9:1, respectively, and so forth. On the other hand, the optimal *α*s seem to vary somewhat, with a value of 0.3 not being too large for most data sets and most percentages of retrieved compounds (see Table 14).

**Table 14:** **Best** ***α* coefficients for the MCS**_***ext***_** and ECFP**_***ext***_** similarity methods (approach B2)**

**DuD set**	**ECFP **_***ext***_			**MCS **_***ext***_		
	**1%**	**5%**	**10%**	**1%**	**5%**	**10%**
HMGR	0.2	0.5	0.9	0.3	0.7	0.8
ER	0.1	0.3	0.2	0.6	0.6	0.5
PPAR *γ*	0.1	0.4	0.0	0.0	0.4	0.0
P38 MAP	0.3	0.5	1.0	0.7	0.8	1.0
TK	0.0	0.1	0.3	0.3	1.0	0.2
Fxa	0.0	0.3	0.2	0.2	0.7	0.6
ADA	0.3	1.0	1.0	0.3	1.0	1.0
DHFR	0.1	0.3	0.5	0.4	1.0	0.6
AchE	0.2	0.3	0.1	0.2	0.3	0.4
COX-2	0.1	0.2	0.2	0.1	1.0	0.6

The best ***α*** coefficients for the MCS_*ext*_ and ECFP_*ext*_ similarity methods. *α* has been increased from 0.0 to 1.0 in steps of 0.1. The coefficient giving the best ***Δ***_*EF*_ value is reported. If two values are identical the smaller ***α*** is reported.

**Table 15:** **Mean** ***Δ***_***EF***_** and standard deviation using the best** ***α* coefficients (approach B1)**

	**MCS **_***ext***_			**ECFP **_***ext***_		
**DuD set**	**1%**	**5%**	**10%**	**1%**	**5%**	**10%**
HMGR	**5.8** ± 10.0	**1.7** ± 0.7	**0.6** ± 0.3	**0.6** ± 1.9	**2.6** ± 3.2	**0.6** ± 0.1
ER	**12.1** ± 6.0	**9.3** ± 3.5	**3.2** ± 1.1	**6.0** ± 5.3	8.5 ± 2.1	**3.1** ± 1.2
PPAR *γ*	**4.5** ± 10.6	3.8 ± 5.5	**1.5** ± 2.9	4.1 ± 10.7	**3.6** ± 5.6	1.7 ± 2.5
P38 MAP	**2.8** ± 6.9	**4.8** ± 4.2	**2.4** ± 2.1	**2.7** ± 6.0	**4.8** ± 4.2	**2.4** ± 2.1
TK	**18.3** ± 5.3	**11.1** ± 3.8	**3.7** ± 1.7	16.5 ± 8.4	**11.1** ± 3.8	**4.2** ± 1.9
FXa	**3.5** ± 11.0	**4.3** ± 5.4	**2.0** ± 2.7	**3.5** ± 11.0	4.2 ± 5.4	2.0 ± 2.7
ADA	**9.2** ± 4.6	**5.2** ± 0.0	**2.2** ± 0.8	**7.8** ± 7.5	**5.2** ± 0.0	**2.2** ± 0.7
DHFR	**2.7** ± 5.1	**0.0** ± 0.0	**0.0** ± 0.0	**1.9** ± 0.9	**0.0** ± 0.0	**0.0** ± 0.0
ACHE	**10.0** ± 11.8	**9.0** ± 6.0	**4.3** ± 2.8	**11.0** ± 12.0	9.0 ± 6.1	**4.0** ± 2.8
COX-2	**9.9** ± 9.8	**9.8** ± 3.7	**2.1** ± 2.6	**6.7** ± 10.2	5.3 ± 5.0	**2.1** ± 2.6
w/d/l	**10** / 0 / 0	**9** / 0 / 1	**10** / 0 / 0	**8** / 0 / 2	**6** / 0 / 4	**8** / 0 / 2

Mean ***Δ***_*EF*_ and standard deviation using the best ***α*** coefficients for extended similarites MCS_*ext*_ and ECFP_*ext*_ for the receptor specific decoy sets DuD_*set*_ at 1%, 5% and 10% of the database. The extension fingerprint is calculated from all ligands (approach B1). Improvements of MCS_*ext*_ compared to MCS as well as ECFP_*ext*_ compared to ECFP are marked in bold print. w/d/l = wins/draws/losses.

**Table 16:** **Mean** ***Δ***_***EF***_** and standard deviation using the best** ***α* coefficients (approach B2)**

	**MCS **_***ext***_			**ECFP **_***ext***_		
**DuD set**	**1%**	**5%**	**10%**	**1%**	**5%**	**10%**
HMGR	**6.1** ± 10.5	**2.1** ± 0.9	**0.8** ± 0.4	**2.7** ± 6.8	**4.3** ± 5.6	**1.5** ± 2.5
ER	**8.1** ± 6.1	**10.4** ± 2.9	**4.6** ± 1.5	**6.3** ± 5.4	10.0 ± 2.0	**4.2** ± 1.3
PPAR *γ*	4.6 ± 10.6	3.9 ± 5.5	1.7 ± 2.8	4.1 ± 10.7	**3.5** ± 5.6	1.7 ± 2.5
P38 MAP	**5.4** ± 6.4	**5.4** ± 3.4	**1.4** ± 0.1	**3.9** ± 5.7	6.7 ± 3.8	**1.4** ± 0.1
TK	**17.4** ± 5.2	**11.4** ± 4.8	**4.7** ± 1.6	16.5 ± 8.4	**11.4** ± 3.5	**4.6** ± 2.1
FXa	**3.5** ± 11.2	**5.7** ± 5.1	**2.5** ± 2.6	**3.5** ± 11.0	5.0 ± 5.2	2.2 ± 2.6
ADA	**9.7** ± 5.4	**7.2** ± 2.6	**2.4** ± 1.1	**7.3** ± 7.2	6.9 ± 2.7	**2.4** ± 1.0
DHFR	**3.0** ± 6.0	**0.8** ± 1.6	**0.0** ± 0.0	**2.4** ± 1.2	**0.4** ± 0.9	**0.0** ± 0.0
ACHE	**10.1** ± 12.0	**9.6** ± 5.8	**4.5** ± 2.9	**11.2** ± 12.2	9.4 ± 5.9	**4.4** ± 2.8
COX-2	**12.0** ± 10.3	**10.8** ± 6.3	**2.8** ± 2.8	**6.7** ± 10.3	5.5 ± 4.9	**2.2** ± 2.6
w/d/l	**9** / 1 / 0	**9** / 0 / 1	**9** / 1 / 0	**8** / 0 / 2	**4** / 0 / 6	**8** / 0 / 2

Mean ***Δ***_*EF*_ and standard deviation using the best ***α*** coefficients for extended similarites MCS_*ext*_ and ECFP_*ext*_ for the receptor specific decoy sets DuD_*set*_ at 1%, 5% and 10% of the database. The extension fingerprint is calculated from 10% (20% for HMGR, TK and ADA) of the ligands (approach B2). Improvements of MCS_*ext*_ compared to MCS as well as ECFP_*ext*_ compared to ECFP are marked in bold print. w/d/l = wins/draws/losses.

To account for the variation of *Δ*_*E* *F*_ across different sets within a cross-validation (see the standard deviations in Tables 8, 9, 10, 11, 12 and 13), we wanted to check whether the scores of two compared methods go up or down in a concerted fashion, or whether this is not the case. For this purpose, we present the win/loss statistics for a fixed *α* of 0.3 in Tables 17 and 18. As can be seen in these tables, the proportion of 8:2 or 9:1 still holds when zooming in on the individual data sets from Tables 8, 9, 11 and 12. Unfortunately, the results are not independent anymore, thus, the sign test can no longer be applied.

**Table 17:** **Win/loss counts for ten random folds for extended similarites on DuD set (*α* =0** ***.*** **3; approach B1)**

	**ECFP **_***ext***_						**MCS **_***ext***_					
	**1%**		**5%**		**10%**		**1%**		**5%**		**10%**	
	**Win**	**Loss**	**Win**	**Loss**	**Win**	**Loss**	**Win**	**Loss**	**Win**	**Loss**	**Win**	**Loss**
HMGR	10	0	10	0	10	0	5	0	5	0	5	0
ER	8	2	10	0	9	1	10	0	10	0	10	0
PPAR *γ*	9	1	9	1	9	1	9	1	10	0	9	1
P38 MAP	9	1	10	0	10	0	10	0	10	0	9	1
TK	10	0	9	1	8	2	9	1	9	1	9	1
FXa	10	0	9	1	9	1	10	0	9	1	9	1
ADA	10	0	6	4	10	0	8	1	8	1	8	1
DHFR	8	2	10	0	10	0	10	0	10	0	10	0
ACHE	9	1	9	1	9	1	10	0	10	0	9	1
COX-2	6	4	7	3	8	2	7	3	9	1	9	1
Sum	89	11	89	11	92	8	88	6	90	4	87	7

Win/loss counts for all ten random folds for extended similarites MCS_*ext*_ and ECFP_*ext*_ versus their base similarities MCS and ECFP for the receptor specific decoy sets DuD_*set*_ at 1%, 5% and 10% of the database. The extension fingerprint is calculated from all ligands (approach B1).

**Table 18:** **Win/loss counts for ten random folds for extended similarites on DuD set (*α* =0** ***.*** **3; approach B2)**

	**ECFP**_***ext***_						**MCS**_***ext***_					
	**1%**		**5%**		**10%**		**1%**		**5%**		**10%**	
	**Win**	**Loss**	**Win**	**Loss**	**Win**	**Loss**	**Win**	**Loss**	**Win**	**Loss**	**Win**	**Loss**
HMGR	10	0	9	1	9	1	5	0	5	0	5	0
ER	8	2	9	1	7	3	10	0	9	1	7	3
PPAR *γ*	10	0	9	1	9	1	6	4	10	0	8	2
P38 MAP	9	1	10	0	9	1	9	1	9	1	10	0
TK	7	3	9	1	9	1	9	1	6	4	7	3
FXa	10	0	7	3	8	2	9	1	8	2	8	2
ADA	10	0	6	4	9	1	6	3	8	1	6	3
DHFR	7	3	10	0	10	0	10	0	10	0	10	0
ACHE	8	2	9	1	9	1	8	2	10	0	9	1
COX-2	8	2	9	1	7	3	7	3	8	2	8	2
Sum	87	13	87	13	86	14	79	15	83	11	78	16

Win/loss counts for all ten random folds for extended similarites MCS_*ext*_ and ECFP_*ext*_ versus their base similarities MCS and ECFP for the receptor specific decoy sets DuD_*set*_ at 1%, 5% and 10% of the database. The extension fingerprint is calculated from 10% (20% for HMGR, TK and ADA) of the ligands (approach B2).

To investigate if the extension similarity sim${}_{\text{bind\_fp}}$ on its own is better than the base similarity measures MCS and ECFP we provide Tables 10 and 13. The results show that the bind_fp similarity in general is not better on its own in comparison to the base similarities. Only for 10% of the database in approach B1 the bind_fp similarity performs better in the ranking than MCS or ECFP.

Our final results on the DuD data sets concern the question whether the method is really sensitive against the choice of a suitable *α*. For this purpose, we present the win/loss statistics for a wide range of *α* values (from 0.0 to 1.0 with a step size of 0.1), across all the data sets from cross-validation in Tables 19 and 20. Quite surprisingly, the choice of a value of *α* does not appear to have a strong influence on the win/loss statistics. The proportion of roughly 8:2 or 9:1 still holds in this experiment. Therefore, we may conclude that the method is reasonably robust regarding the choice of a suitable value for *α*.

**Table 19:** **Win/loss counts for all random folds for extended similarites on DuD set (*α*** ***∈* (0.0, 0.1); approach B1)**

	**ECFP **_***ext***_						**MCS **_***ext***_					
	**1%**		**5%**		**10%**		**1%**		**5%**		**10%**	
	**Win**	**Loss**	**Win**	**Loss**	**Win**	**Loss**	**Win**	**Loss**	**Win**	**Loss**	**Win**	**Loss**
HMGR	102	8	110	0	110	0	48	7	51	4	55	0
ER	70	40	98	12	99	11	97	13	109	1	103	7
PPAR *γ*	93	17	78	32	86	24	94	16	90	20	77	33
P38 MAP	102	8	104	6	104	6	103	7	105	5	100	10
TK	96	14	99	11	92	18	96	14	97	13	99	11
FXa	101	9	100	10	94	16	104	6	100	10	100	10
ADA	91	19	89	21	109	1	81	18	96	3	84	15
DHFR	74	36	110	0	110	0	104	6	110	0	110	0
ACHE	93	17	100	10	101	9	96	14	105	5	101	9
COX-2	57	53	60	50	90	20	78	32	86	24	100	10
Sum	879	221	948	152	995	105	901	133	949	85	929	105

Win/loss counts for all 110 random folds (10 repetitions * 11 ***α*** s) for extended similarites MCS_*ext*_ and ECFP_*ext*_ versus their base similarities MCS and ECFP for the receptor specific decoy sets DuD_*set*_ at 1%, 5% and 10% of the database. The extension fingerprint is calculated from all ligands (approach B1).

**Table 20:** **Win/loss counts for all random folds for extended similarites on DuD set (*α*** ***∈* (0.0, 0.1); approach B2)**

	**ECFP **_***ext***_						**MCS **_***ext***_					
	c**1%**		**5%**		**10%**		**1%**		**5%**		**10%**	
	**Win**	**Loss**	**Win**	**Loss**	**Win**	**Loss**	**Win**	**Loss**	**Win**	**Loss**	**Win**	**Loss**
HMGR	102	8	88	22	98	12	48	7	49	6	55	0
ER	70	40	74	36	74	36	87	23	89	21	67	43
PPAR *γ*	93	17	86	24	88	22	69	41	95	15	72	38
P38 MAP	93	17	96	14	103	7	93	17	100	10	110	0
TK	75	35	95	15	94	16	96	14	85	25	82	28
FXa	101	9	67	43	80	30	99	11	84	26	83	27
ADA	86	24	79	31	98	12	60	39	88	11	76	23
DHFR	75	35	107	3	110	0	100	10	110	0	110	0
ACHE	86	24	95	15	96	14	87	23	98	12	94	16
COX-2	69	41	76	34	78	32	65	45	86	24	90	20
Sum	850	250	863	237	919	181	804	230	884	150	839	195

Win/loss counts for all 110 random folds (10 repetitions * 11 ***α*** s) for extended similarites MCS_*ext*_ and ECFP_*ext*_ versus their base similarities MCS and ECFP for the receptor specific decoy sets DuD_*set*_ at 1%, 5% and 10% of the database. The extension fingerprint is calculated from 10% (20% for HMGR, TK and ADA) of the ligands (approach B2).

Comparing the data mining based extension results for the HMGR data set (first rows denoted HMGR in Tables 8 and 9) with the by-hand results on HMGR in Table 6 (rows denoted “lig vs DuD _*s* *e* *t*_”), we see that the *Δ*_*E* *F*_ values are slightly better for the by-hand extension, but both variants of the data mining based approach are quite competitive. The ECFP _*e* *x* *t*_ results of variant B1 are even better than the by-hand results.

As final experiments to test our data-mining based approaches B1 and B2 we added 25 ChEMBL activity class data sets. The results for approach B1 and B2 are given in Tables 21 and 22 respectively. For those data sets the win counts over all data sets are 19, 21, 21 and 18, 22, 22 (of 25 maximum possible) for 1%, 5% and 10% of the database and MCS _*e* *x* *t*_ and ECFP _*e* *x* *t*_. According to the sign test the difference between extended and non-enxtended similarities is significant at a level of 0.05 [22].

**Table 21:** **Mean** ***Δ***_***EF***_** and standard deviation for the experiments on the ChEMBL activity classes (approach B2)**

	**MCS**			**MCS **_***ext***_		
**CAC**	**1%**	**5%**	**10%**	**1%**	**5%**	**10%**
4	50.0 ± 23.1	5.8 ± 5.5	2.2 ± 2.6	**40.0** ± 11.1	**4.0** ± 3.2	**0.0** ± 1.5
9	66.7 ± 14.3	13.3 ± 2.9	6.5 ± 1.4	**59.3** ± 17.2	**11.1** ± 5.0	**5.6** ± 2.8
10	66.0 ± 16.5	13.2 ± 3.3	6.2 ± 1.6	**53.3** ± 9.8	**6.7** ± 1.5	**2.7** ± 0.9
21	80.0 ± 8.2	15.4 ± 2.3	7.4 ± 1.4	80.0 ± 15.8	16.0 ± 2.6	8.0 ± 0.0
35	75.0 ± 9.6	14.2 ± 1.7	7.1 ± 0.8	**72.2** ± 11.7	**12.2** ± 4.8	**1.1** ± 3.2
44	66.4 ± 25.8	9.3 ± 6.3	3.3 ± 3.2	**30.1** ± 21.1	**6.0** ± 3.5	**3.0** ± 1.7
52	71.4 ± 9.8	13.0 ± 2.1	5.6 ± 0.9	**70.0** ± 9.7	**4.0** ± 0.8	**0.0** ± 1.0
54	81.4 ± 10.7	15.4 ± 2.5	7.4 ± 1.5	**70.0** ± 16.6	**12.1** ± 0.0	**0.0** ± 0.0
57	54.0 ± 23.2	9.2 ± 5.7	4.4 ± 2.8	60.0 ± 13.5	**3.9** ± 6.9	4.9 ± 1.1
81	82.0 ± 7.9	15.4 ± 1.6	6.7 ± 0.9	**80.0** ± 6.7	**9.8** ± 1.5	**2.0** ± 2.6
86	67.0 ± 18.3	10.2 ± 5.4	4.0 ± 3.0	**50.0** ± 17.9	**4.0** ± 0.7	5.1 ± 1.4
98	80.0 ± 10.0	14.7 ± 2.4	6.7 ± 1.5	**70.0** ± 14.6	**6.1** ± 4.9	**3.0** ± 1.8
105	72.5 ± 14.9	13.3 ± 4.3	6.6 ± 2.1	88.7 ± 10.1	**10.0** ± 2.5	**2.9** ± 2.6
113	71.1 ± 7.8	12.9 ± 1.1	5.9 ± 0.6	**70.1** ± 6.9	**8.4** ± 3.1	**1.0** ± 1.4
121	74.0 ± 5.2	14.8 ± 1.0	7.2 ± 0.6	**69.9** ± 4.9	**13.9** ± 1.2	**6.9** ± 1.4
129	65.0 ± 10.0	10.5 ± 1.0	4.8 ± 1.3	**50.0** ± 9.9	**2.0** ± 0.4	**0.9** ± 1.6
152	80.0 ± 12.2	16.0 ± 2.4	8.0 ± 1.2	**76.5** ± 17.6	16.0 ± 0.6	8.0 ± 2.6
181	66.0 ± 5.5	9.6 ± 2.6	2.8 ± 0.8	**60.0** ± 7.6	**4.0** ± 4.7	**0.8** ± 1.6
186	80.0 ± 7.1	14.0 ± 2.8	6.0 ± 1.6	**20.0** ± 15.6	**2.0** ± 3.9	**0.0** ± 1.8
195	77.8 ± 11.1	14.7 ± 2.5	5.8 ± 0.9	**62.9** ± 4.5	**9.6** ± 3.6	**4.8** ± 2.6
211	50.0 ± 0.0	10.0 ± 0.0	5.0 ± 0.0	50.0 ± 18.0	**0.0** ± 3.7	**0.0** ± 1.6
213	77.8 ± 13.6	15.1 ± 2.4	7.1 ± 1.5	**74.1** ± 6.3	**13.3** ± 0.8	**6.7** ± 2.6
230	64.0 ± 19.5	8.4 ± 6.1	3.4 ± 2.9	90.0 ± 20.1	14.0 ± 1.6	**0.9** ± 1.4
234	52.0 ± 16.4	10.4 ± 3.3	5.2 ± 1.6	**40.0** ± 15.5	**6.2** ± 2.7	**1.0**s ± 1.6
238	66.0 ± 15.2	10.4 ± 4.3	4.8 ± 2.0	70.0 ± 12.0	14.0 ± 4.9	**3.1** ± 1.8
w/d/l				**19** / 2 / 4	**21** / 1 / 3	**21** / 1 / 3

Mean ***Δ***_*EF*_ and standard deviation for the MCS and MCS_*ext*_ similarity methods at 1%, 5% and 10% of the database (ZINC subset). The extension fingerprint is calculated from 10% of the ligands (approach B2). Improvements of MCS_*ext*_ compared to MCS are marked in bold print. CAC = ChEMBL activity class. w/d/l = wins/draws/losses.

**Table 22:** **Mean** ***Δ***_***EF***_** and standard deviation for the experiments on the ChEMBL activity classes (approach B2)**

	**ECFP**			**ECFP **_***ext***_		
**CAC**	**1%**	**5%**	**10%**	**1%**	**5%**	**10%**
4	46.0 ± 17.1	6.2 ± 3.7	1.6 ± 1.3	**30.0** ± 9.9	**4.0** ± 4.9	**0.0** ± 0.0
9	65.4 ± 13.0	12.1 ± 2.5	5.3 ± 1.6	**59.3** ± 9.7	**11.1** ± 4.7	**5.2** ± 3.2
10	66.0 ± 11.7	11.0 ± 3.3	4.9 ± 1.5	**46.7** ± 10.3	**6.1** ± 3.9	**1.9** ± 1.3
21	66.0 ± 17.8	12.2 ± 3.7	5.8 ± 1.8	**49.9** ± 17.8	**8.0** ± 2.5	**3.0** ± 0.0
35	44.4 ± 25.1	6.7 ± 6.3	2.8 ± 2.7	66.7 ± 11.6	**5.6** ± 2.3	**0.0** ± 1.3
44	70.0 ± 12.5	12.8 ± 3.3	5.7 ± 1.7	**39.4** ± 14.3	**8.0** ± 2.5	**4.1** ± 1.8
52	71.0 ± 11.0	11.0 ± 2.4	4.4 ± 1.1	**39.6** ± 16.5	**0.0** ± 3.3	**0.0** ± 0.8
54	74.0 ± 11.7	12.4 ± 4.1	5.3 ± 1.9	**60.0** ± 10.5	**7.6** ± 1.6	**1.9** ± 1.5
57	59.0 ± 17.3	10.0 ± 3.9	3.9 ± 2.1	**50.0** ± 12.5	**2.0** ± 0.9	**0.8** ± 2.2
81	77.0 ± 6.7	14.2 ± 1.5	6.5 ± 1.0	80.0 ± 11.0	**12.0** ± 2.6	**3.6** ± 1.5
86	55.0 ± 17.2	7.4 ± 4.4	2.6 ± 2.0	70.0 ± 11.7	**6.0** ± 4.8	**1.0** ± 1.8
98	60.0 ± 22.1	11.2 ± 4.3	5.5 ± 2.3	**40.0** ± 25.8	**6.0** ± 3.3	**3.0** ± 1.0
105	58.0 ± 24.9	10.8 ± 4.6	5.2 ± 2.2	60.0 ± 9.8	**10.0** ± 2.4	**4.0** ±s 0.0
113	64.0 ± 10.8	10.0 ± 3.4	4.2 ± 1.8	**50.0** ± 7.9	**2.1** ± 2.6	**0.9** ± 2.6
121	74.0 ± 5.2	14.6 ± 1.0	6.5 ± 0.8	**70.0** ± 10.7	**14.0** ± 2.4	6.8 ± 1.6
129	69.0 ± 9.9	12.4 ± 1.6	5.5 ± 1.5	**49.8** ± 23.2	**6.0** ± 4.3	**0.8** ± 1.3
152	74.0 ± 12.6	14.6 ± 2.5	6.9 ± 1.5	90.0 ± 13.3	**14.2** ± 0.7	**4.2** ± 1.7
181	61.0 ± 12.9	10.6 ± 3.0	4.7 ± 1.6	**39.8** ± 24.9	**7.8** ± 4.9	**3.0** ± 1.9
186	60.0 ± 14.9	7.4 ± 4.6	2.6 ± 1.7	**20.1** ± 13.6	**2.0** ± 2.5	**0.0** ± 2.1
195	69.1 ± 14.5	12.3 ± 2.7	5.7 ± 1.4	**59.3** ± 19.5	**11.1** ± 3.3	**4.8** ± 1.8
211	42.0 ± 9.2	7.8 ± 2.9	3.1 ± 1.4	**19.8** ± 12.6	**0.0** ± 1.2	**0.0** ± 2.6
213	61.7 ± 13.7	9.4 ± 2.2	3.7 ± 1.1	**55.6** ± 20.2	10.4 ± 0.4	5.2 ± 1.4
230	60.0 ± 18.3	10.2 ± 3.6	3.3 ± 1.3	90.0 ± 17.8	11.9 ± 3.6	**2.0** ± 1.4
234	57.0 ± 17.0	8.4 ± 3.9	3.2 ± 1.7	**41.2** ± 14.9	**2.0** ± 4.7	**1.1** ± 1.6
238	64.0 ± 17.8	11.8 ± 3.9	5.0 ± 1.9	80.0 ± 14.5	16.3 ± 1.6	7.0 ± 1.8
w/d/l				**18** / 0 / 7	**22** / 0 / 3	**22** / 0 / 3

Mean ***Δ***_*EF*_ and standard deviation for the ECFP and ECFP_*ext*_ similarity methods at 1%, 5% and 10% of the database (ZINC subset). The extension fingerprint is calculated from 10% of the ligands (approach B2). Improvements of MCS_*ext*_ compared to MCS are marked in bold print. CAC = ChEMBL activity class. w/d/l = wins/draws/losses.

## Conclusions

Structural similarity measures, especially the ECFP fingerprints, have been reported to be superior to non-substructural fingerprints [23]. This work shows that and how such structural similarity methods used in virtual screening can be improved further by integrating background knowledge on binding-relevant structural features. We presented an approach based on by-hand selection of the background knowledge as well as an approach working with fragment-based data mining. From our experimental evaluation we conclude that the addition of only one binding-relevant sub-structural feature of a known ligand can substantial improve the enrichment factors in the virtual screening. We additionally show that using data mining based knowledge extraction instead of time consuming by-hand selection of relevant features gives competitive results.

## Competing interests

The authors declare that they have no competing interests.

## Authors’ contributions

TG implemented the similarity methods designed and carried out the experiments and evaluation and wrote the manuscript. LP did the docking experiments and contributed to the manuscript. SK helped to draft the manuscript and contributed to the experimental design and setup. All authors read and approved the final manuscript.

## Supplementary Material

Additional file 1 **Supplementary material for improving structural similarity based virtual screening using background knowledge.** The supplementary information contains more extensive result tables and additional mathematical equations. Click here for file

## References

[B1] Terstappen G Reggiani A **In silico research in drug discovery** Trends Pharmacol Sci 2001 5 23 26 11165668 10.1016/s0165-6147(00)01584-4

[B2] van de Waterbeemed H Gifford E **ADMET in silico modelling: towards prediction paradise?** Nat Rev Drug Discov 2003 5 192 204 10.1038/nrd1032 12612645

[B3] Rückert U Kramer S **Frequent free tree discovery in graph data** Proceedings of the ACM SIG Symposium on Applied Computing (SAC’04) 2004 New York, NY, USA: ACM Press 564 570

[B4] Raymond J Gardiner E Willett P **RASCAL: calculation of graph similarity using maximum common edge subgraphs** Comput J 2002 5 6 631 644 10.1093/comjnl/45.6.631 11911700

[B5] Rogers D Hahn M **Extended-connectivity fingerprints** J Chem Inf Model 2010 5 5 742 754 10.1021/ci100050t 20426451

[B6] Wallis W Shoubridge P Kraetz M Ray D **Graph distances using graph union** Pattern Recognit Lett 2001 5 701 704 [http://dx.doi.org/10.1016/S0167-8655(01)00022-8] 10.1016/S0167-8655(01)00022-8

[B7] Weininger D Weininger A Weininger J **SMILES. 2. algorithm for generation of unique SMILES notation** J Chem Inf Comput Sci 1989 5 2 97 101 10.1021/ci00062a008

[B8] Stalring J Carlsson L Almeida P Boyer S **AZOrange-High performance open source machine learning for QSAR modeling in a graphical programming environment** J Cheminformatics 2011 5 28 10.1186/1758-2946-3-28 PMC3158423 21798025

[B9] Knox C Law V Jewison T Liu P Ly S Frolkis A Pon A Banco K Mak C Neveu V Djoumbou Y Eisner R Guo AC Wishart DS **DrugBank 3.0: a comprehensive resource for ‘Omics’ research on drugs** Nucl Acids Res 2011 5 suppl 1 D1035 D1041 21059682 10.1093/nar/gkq1126 PMC3013709

[B10] Huang N Shoichet B Irwin J **Benchmarking sets for molecular docking** J Med Chem 2006 5 23 6789 6801 10.1021/jm0608356 17154509 PMC3383317

[B11] Heikamp K Bajorath J **Large-scale similarity search profiling of ChEMBL compound data sets** J Chem Inf Model 2011 5 8 1831 1839 10.1021/ci200199u 21728295

[B12] Irwin JJ Sterling T Mysinger MM Bolstad ES Coleman RG **ZINC: a free tool to discover chemistry for biology** J Chem Inf Model 2012 5 7 1757 1768 10.1021/ci3001277 22587354 PMC3402020

[B13] Lewington S Whitlock G Clarke R Sherliker P Emberson J Halsey J Qizilbash N Peto R Collins R **Blood cholesterol and vascular mortality by age, sex, and blood pressure: a meta-analysis of individual data from 61 prospective studies with 55000 vascular deaths** The Lancet 2007 5 9602 1829 1839 10.1016/S0140-6736(07)61778-4 18061058

[B14] Eisenberg D **Cholesterol lowering in the management of coronary artery disease: the clinical implications of recent trials** Am J Med 1998 5 2, Supplement 1 2S 5S 10.1016/S0002-9343(98)00038-2 9550499

[B15] Endo A Kuroda M Tanzawa K **Competitive inhibition of 3-hydroxy-3-methylglutaryl coenzyme A reductase by ML-236A and ML-236B fungal metabolites, having hypocholesterolemic activity** FEBS Lett 1976 5 2 323 326 10.1016/0014-5793(76)80996-9 16386050

[B16] Berman H Westbrook J Feng Z Gilliland G Bhat T Weissig H Shindyalov I Bourne P **The protein data bank** Nucl Acids Res 2000 5 235 242 10.1093/nar/28.1.235 10592235 PMC102472

[B17] Istvan E Deisenhofer J **Structural mechanism for statin inhibition of HMG-CoA reductase** Science 2001 5 5519 1160 1164 [http://www.sciencemag.org/content/292/5519/1160.abstract] 10.1126/science.1059344 11349148

[B18] Scarsi M Podvinec M Roth A Hug H Kersten S Albrecht H Schwede T Meyer UA Ruecker C **Sulfonylureas and Glinides exhibit peroxisome proliferator-activated receptor gamma activity: A combined virtual screening and biological assay approach** Mol Pharmacol 2007 5 2 398 406 17082235 10.1124/mol.106.024596

[B19] Bemis GW Murcko MA **The properties of known drugs. 1. Molecular frameworks** J Med Chem 1996 5 15 2887 2893 10.1021/jm9602928 8709122

[B20] Evers A Klabunde T **Structure-based drug discovery using GPCR homology modeling: successful virtual screening for antagonists of the alpha1A adrenergic receptor** J Med Chem 2005 5 4 1088 1097 10.1021/jm0491804 15715476

[B21] Liberato MV Nascimento AS Ayers SD Lin JZ Cvoro A Silveira RL Martínez L Souza PCT Saidemberg D Deng T Amato AA Togashi M Hsueh WA Phillips K Palma MS Neves FAR Skaf MS Webb P Polikarpov I **Medium chain fatty acids are selective peroxisome proliferator activated receptor (PPAR) gamma activators and Pan-PPAR partial agonists** PLoS ONE 2012 5 5 e36297 10.1371/journal.pone.0036297 22649490 PMC3359336

[B22] Demšar J **Statistical comparisons of classifiers over multiple data sets** J Mach Learn Res 2006 5 1 30

[B23] Hert J Willett P Wilton DJ Acklin P Azzaoui K Jacoby E Schuffenhauer A **Comparison of topological descriptors for similarity-based virtual screening using multiple bioactive reference structures** Org Biomol Chem 2004 5 3256 3266 10.1039/b409865j 15534703

