# Supplementary material for: Improving structural similarity based virtual screening using background knowledge
Source: J Cheminform. 2013 Dec 16;5:50. doi: 10.1186/1758-2946-5-50 (PMC3928642; doi:10.1186/1758-2946-5-50)
Supplement: Additional file 1 — Supplementary material for improving structural similarity based virtual screening using background knowledge. The supplementary information contains more extensive result tables and additional mathematical equations. [file 1758-2946-5-50-S1.pdf]

# Supplementary Material for Improving Structural Similarity Based Virtual Screening Using Background Knowledge

Tobias Girschick[1] and Lucia Puchbauer[1] and Stefan Kramer*[2]

[1]Technische Universität München, Institut für Informatik, Boltzmannstrasse 3, 85748 Garching b. München, Germany
[2]Johannes Gutenberg-Universität Mainz, Institut für Informatik, Staudingerweg 9, 55128 Mainz, Germany

Email: Tobias Girschick - tobias.girschick@in.tum.de; Lucia Puchbauer - puchbaue@in.tum.de; Stefan Kramer*-kramer@informatik.uni-mainz.de;

*Corresponding author

## Mathematical Equations

### Dice coefficient

$$sim_{Dice}(x,y) = \frac{2c}{a+b}, \quad (1)$$

where $a$, $b$ and $c$ are defined as in the Tanimoto coefficient. Note, that the Dice coefficient is monotonic with the Tanimoto coefficient.

### Tanimoto coefficient

$$sim_{Tanimoto}(x,y) = \frac{c}{a+b-c}, \quad (2)$$

where $a$ and $b$ is the number of bits set to 1 in molecules $x$ and $y$, and $c$ the number of bits set to 1 in both, $x$ and $y$.

## Additional Tables

| Rank | CID | Docking Score | $\text{Rank}_{MCS}$ | $\Delta_{Rank_{MCS}}$ |
|---|---|---|---|---|
| 1 | 60823 | -10.564 | 2 | -1 |
| 2 | ZINC02336737 | -5.808526 | 13 | -11 |
| 3 | ZINC00026851 | -5.699634 | 19 | -16 |
| 4 | ZINC00588719 | -5.568737 | 11 | -7 |
| 5 | ZINC00599752 | -5.46502 | 5 | 0 |
| 6 | ZINC00588053 | -5.463745 | 16 | -10 |
| 7 | ZINC00864379 | -5.291673 | 15 | -8 |
| 8 | ZINC01253780 | -5.211104 | 14 | -6 |
| 9 | ZINC00714466 | -5.149133 | 9 | 0 |
| 10 | ZINC00588723 | -5.14689 | 4 | 6 |
| 11 | ZINC00590911 | -5.135349 | 25 | -14 |
| 12 | ZINC04128931 | -5.101469 | 22 | -10 |
| 13 | ZINC01032240 | -5.094384 | 8 | 5 |
| 14 | ZINC00658975 | -5.038167 | 20 | -6 |
| 15 | ZINC00590434 | -4.973652 | 21 | -6 |
| 16 | ZINC00625939 | -4.972097 | 18 | -2 |
| 17 | ZINC01112466 | -4.918515 | 10 | 7 |
| 18 | ZINC04628438 | -4.916212 | 7 | 11 |
| 19 | ZINC02049068 | -4.914307 | 17 | 2 |
| 20 | ZINC00803728 | -4.669317 | 24 | -4 |
| 21 | ZINC03273040 | -4.569581 | 23 | -2 |
| 22 | 24848419 | -4.425088 | 3 | 19 |
| 23 | ZINC03837410 | -4.29318 | 12 | 11 |
| 24 | ZINC00588941 | -4.144152 | 26 | -2 |
| 25 | ZINC02129514 | -4.095075 | 6 | 19 |

Table S1: Results of the first docking run. $\Delta_{Rank} = Rank_{docking} - Rank_{MCS}$. A negative $\Delta_{Rank}$ value means, in the MCS similarity the compound is ranked lower, a positive $\Delta_{Rank}$ that it is ranked higher than by the docking procedure.

| Rank | CID | Score | $\text{Rank}_{MCS_{ext}}$ | $\Delta_{Rank_{MCSext}}$ |
|---|---|---|---|---|
| 1 | ZINC00588723 | -10.382184 | 16 | -15 |
| 2 | 24848419 | -7.980885 | 3 | -1 |
| 3 | ZINC01253780 | -7.385909 | 9 | -6 |
| 4 | ZINC00625939 | -7.157018 | 11 | -7 |
| 5 | ZINC01032240 | -7.104563 | 5 | 0 |
| 6 | ZINC00864379 | -7.052449 | 10 | -4 |
| 7 | ZINC00026851 | -6.910078 | 19 | -12 |
| 8 | ZINC00714466 | -6.702119 | 6 | 2 |
| 9 | ZINC01112466 | -6.667553 | 7 | 2 |
| 10 | 64715 | -6.654007 | 12 | -2 |
| 11 | ZINC02336737 | -6.537559 | 8 | 3 |
| 12 | ZINC00590911 | -6.45151 | 21 | -9 |
| 13 | 60823 | -6.29428 | 2 | 11 |
| 14 | ZINC03431465 | -6.289821 | 26 | -12 |
| 15 | ZINC00599752 | -6.09275 | 4 | 11 |
| 16 | ZINC00588053 | -5.887202 | 22 | -6 |
| 17 | ZINC04259960 | -5.79234 | 20 | -3 |
| 18 | ZINC02563245 | -5.748378 | 24 | -6 |
| 19 | 53232 | -5.683409 | 13 | 6 |
| 20 | ZINC03202042 | -5.606497 | 15 | 5 |
| 21 | ZINC04597014 | -5.130039 | 23 | -2 |
| 22 | ZINC03639638 | -5.095658 | 17 | 5 |
| 23 | ZINC03671410 | -4.205335 | 18 | 5 |
| 24 | 54454 | -3.865563 | 14 | 10 |
| 25 | ZINC02129514 | -3.838221 | 25 | 0 |

Table S2: Results of the second docking run. $\Delta_{Rank} = Rank_{docking} - Rank_{MCSorECFP}$. A negative $\Delta_{Rank}$ value means, in the extended similarity the compound is ranked lower, a positive $\Delta_{Rank}$ that it is ranked higher than by the docking procedure.
